# An Information-theoretic framework for the peloric *Lcyc* epiallele in *Linaria vulgaris*: a synthetic demonstration

**DOI:** 10.3389/fpls.2026.1915131

**Published:** 2026-10-16

**Authors:** Ildefonso M. De la Fuente, José Carrasco-Pujante, Leire Legarreta, Iker Malaina, Gorka Pérez-Yarza, Luis Martínez, Jesús M. Cortés, José I. López

**Affiliations:** 1 Department of Nutrition, Centro de Edafología y Biología Aplicada del Segura, Consejo Superior de Investigaciones Científicas (Spanish National Research Council) (CEBAS-CSIC) Institute, Murcia, Spain; 2 Department of Mathematics, Faculty of Science and Technology, University of the Basque Country, Leioa, Spain; 3 Department of Cell Biology and Histology, Faculty of Medicine and Nursing, University of the Basque Country, Leioa, Spain; 4 Biobizkaia Health Research Institute, Barakaldo, Spain; 5 Ikerbasque: The Basque Foundation for Science, Bilbao, Spain

**Keywords:** DNA methylation, epiallele, epigenetic information, floral symmetry, Jensen-Shannon divergence, Kullback–Leibler divergence, *Linaria vulgaris*, peloria

## Abstract

**Introduction:**

The peloric form of *Linaria vulgaris*, first described by Linnaeus in the eighteenth century, is a classical example of heritable epigenetic variation associated with a major change in floral morphology. Wild-type flowers exhibit bilateral symmetry, whereas peloric flowers exhibit radial symmetry. This transition is associated with transcriptional silencing and increased DNA methylation of Lcyc, a CYCLOIDEA-like gene involved in dorsoventral floral development. Although the molecular association between the Lcyc epiallelic state and floral architecture is well established, a general framework for expressing differences between such methylation states in information-theoretic units has not been developed.

**Methods:**

We developed an information-theoretic framework in which potentially methylatable cytosines are represented as Bernoulli variables. Because experimental cytosine-level count data for the natural *Lcyc* epiallele were not available for direct reanalysis, the numerical demonstration uses a synthetic, literature-constrained methylation matrix designed to reproduce the qualitative pattern reported for wild-type and peloric *Lcyc*. The framework quantifies maximum binary capacity, summed site-wise Shannon entropy, directional Kullback–Leibler divergence, summed site-wise Jensen–Shannon divergence, context-specific contributions, and site-wise dimensionality.

**Results:**

In the synthetic demonstration matrix, maximum binary capacity was 120 bits, summed site-wise entropy was 39.85 bits for the modeled wild-type state and 101.32 bits for the modeled peloric state, directional Kullback–Leibler divergence was 260.30 bits, and summed site-wise Jensen–Shannon divergence was 42.31 bits. These numerical values characterize the illustrative matrix under its specified assumptions and should not be interpreted as empirical estimates of the natural *Lcyc* epiallele. Rather, they demonstrate how cytosine-level methylation profiles can be translated into measures of uncertainty, statistical distinguishability, methylation-context contribution, and the distribution of divergence across a locus.

## 1 Introduction

The relationship between genetic information, epigenetic regulation, and biological form remains one of the central problems in evolutionary and developmental biology (Richards, 2006; Weigel and Colot, 2012). In plants, DNA methylation constitutes a heritable molecular regulatory system capable of modifying gene expression without necessarily altering the primary nucleotide sequence (Law and Jacobsen, 2010; He et al., 2011; Quadrana and Colot, 2016). Unlike in animals, where DNA methylation is mainly concentrated at CpG sites, plants maintain methylation in three sequence contexts: CG, CHG, and CHH, where H represents A, C, or T (Law and Jacobsen, 2010; He et al., 2011). This contextual diversity allows plant genomes to store and transmit complex regulatory states across cell divisions and, in some cases, across generations (Richards, 2006; Quadrana and Colot, 2016).

A paradigmatic example of natural epigenetic variation is the peloric form of *Linaria vulgaris*. Wild-type plants produce bilaterally symmetric flowers, whereas peloric plants develop radially symmetric flowers (Cubas et al., 1999). Molecular analysis of this phenotype identified *Lcyc*, a homolog of *CYCLOIDEA*, as a central component in the establishment of dorsoventral floral symmetry (Cubas et al., 1999). In peloric plants, *Lcyc* is transcriptionally silenced and associated with extensive DNA methylation (Cubas et al., 1999; Ehrenhofer-Murray, 2021). Partial or somatic phenotypic reversion correlates with demethylation of the locus and restoration of *Lcyc* expression, suggesting that the epigenetic state of *Lcyc* functions as a heritable regulatory switch (Cubas et al., 1999).

Despite the historical and conceptual importance of this example, the informational magnitude of the peloric *Lcyc* epiallele has not been formally quantified. Shannon’s mathematical theory (Shannon, 1948) provides a natural language for this purpose, allowing uncertainty, coding capacity, and differences between state distributions to be expressed in bits, and the present study is methodological rather than an empirical measurement of the natural Lcyc epiallele. The numerical implementation is therefore intended as a proof-of-principle demonstration using a synthetic matrix constrained by published biological observations.

Specifically, we develop a framework for characterizing the methylation-state information associated with the peloric Lcyc demonstration, and the framework quantifies five related quantities. First, it defines the maximum binary capacity of the assayed region as the number of potentially methylatable cytosines analyzed in CG, CHG, and CHH contexts. Second, it estimates Shannon entropy from site-specific methylation probabilities, thereby quantifying methylation uncertainty or heterogeneity within wild-type and peloric states. Third, it measures informational divergence between wild-type and peloric methylation profiles using Bernoulli Kullback-Leibler divergence. Fourth, it quantifies symmetric marginal separation using the summed site-wise Jensen–Shannon divergence. Fifth, it evaluates the dimensionality of the epigenetic transition by determining whether divergence is concentrated in a small number of cytosines or distributed across a broader regional methylation architecture.

When applied to the synthetic literature-constrained demonstration matrix used in this study, the framework yields a maximum capacity of 120 bits, with modeled wild-type and peloric entropies of 39.85 and 101.32 bits, respectively. These values describe the behavior of the framework under the specified assumptions and should not be interpreted as empirical measurements of the natural Lcyc epiallele.

Throughout this study, the term information is used in the Shannon-theoretic statistical sense: the proposed quantities describe uncertainty and statistical distinguishability of methylation-state distributions, rather than functional biological information or causal contribution to floral morphology.

The peloric form of *Linaria vulgaris*, first observed by Linnaeus in 1742 and subsequently described in the eighteenth-century botanical literature (Gustafsson, 1979), provides a classical example of how epigenetic information is associated with a visible morphological phenotype. The extensive methylation and transcriptional silencing of the Lcyc gene (a CYCLOIDEA-like regulator) is associated with disruption of dorsoventral floral identity, resulting in a shift from bilateral to radial floral symmetry (Cubas et al., 1999; Ehrenhofer-Murray, 2021). The biological rationale for treating the peloric Lcyc epiallele as a regional methylation state is summarized in **Figure 1**. This epigenetic phenotype has persisted in botanical observation for more than two centuries, the longest observed transmission period of a heritable epigenetic phenotype. In the present framework, such historical methylation system is used to ask how the separation between wild-type and peloric Lcyc methylation states can be expressed quantitatively in bits.

**Figure 1:**
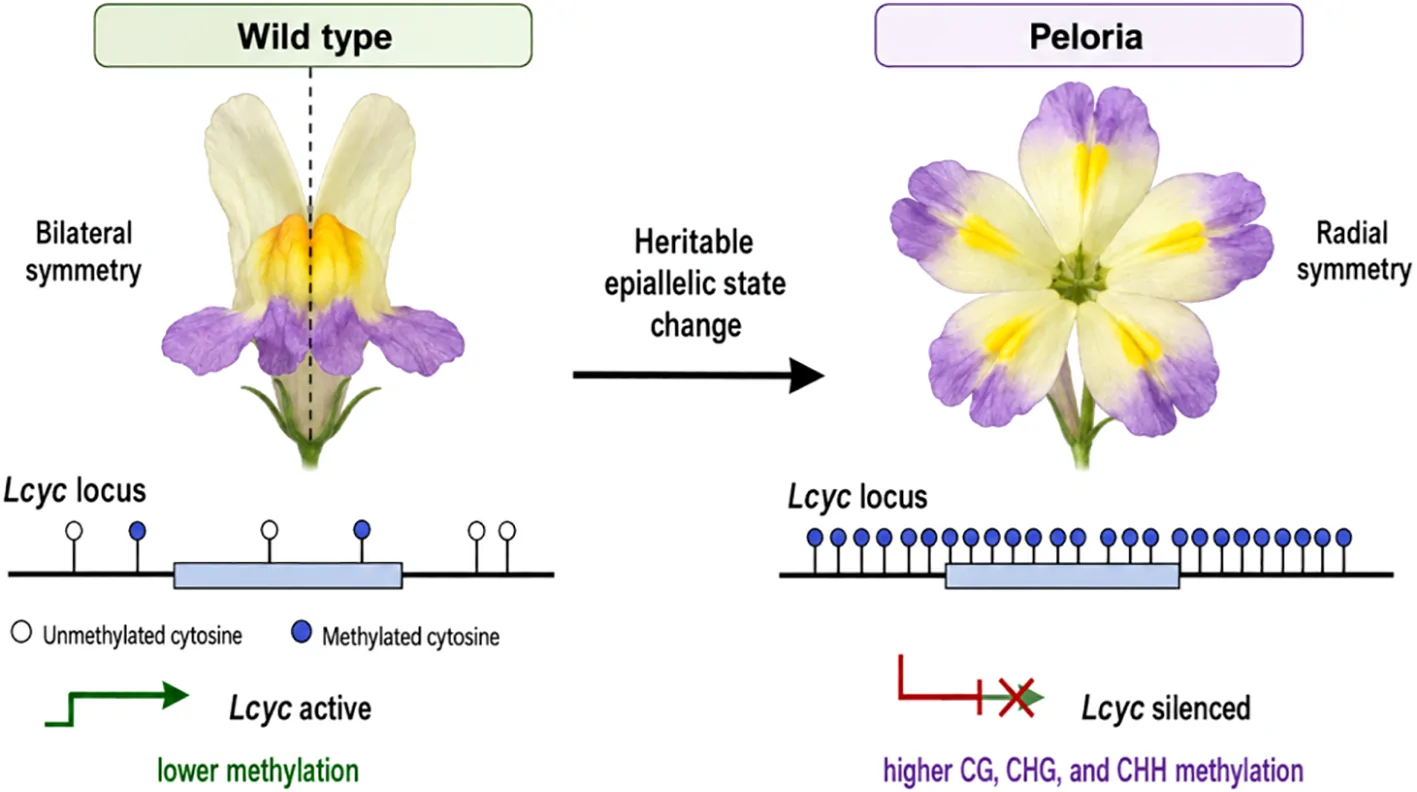
Biological basis of the peloric *Lcyc* epiallele. Conceptual comparison between the wild-type and peloric floral states of *Linaria vulgaris*. Wild-type flowers exhibit bilateral symmetry and are associated with an active *Lcyc* locus showing relatively low methylation. In contrast, peloric flowers exhibit radial symmetry and are associated with transcriptional silencing of *Lcyc* and increased methylation across CG, CHG, and CHH sequence contexts. The figure summarizes the biological rationale for treating the peloric *Lcyc* epiallele as a heritable regional methylation state. Five main information-theoretic quantities are considered in this study: maximum binary capacity, Shannon entropy, directional divergence, summed site-wise Jensen–Shannon divergence, and dimensionality.

To demonstrate that the framework is not restricted to the synthetic Lcyc matrix, we additionally applied the same analytical procedure to an independent publicly available experimental cytosine-level bisulfite-sequencing dataset from the Arabidopsis thaliana FWA locus; this empirical demonstration is presented in the Supplementary Information.

In summary, the use of Shannon entropy and information-theoretic divergence to characterize DNA methylation is established; the specific contribution of this work is the integration of maximum binary capacity, site-wise entropy, directional and symmetric divergence, methylation-context decomposition, and divergence-based dimensionality within a single framework applied to a defined epiallelic transition. It allows the classical peloric mutant of *Linaria vulgaris* to be reconsidered not only as a historical example of epigenetic inheritance, but also as a model for asking how distributed molecular regulatory states can be expressed, compared, and eventually measured in bits.

## 2 Framework and reproducibility methods

### 2.1 Biological rationale and literature constraints

This study develops a quantitative framework based on Shannon’s Information Theory to estimate the methylation-state information associated with the peloric *Lcyc* epiallele in *Linaria vulgaris*. Accordingly, the numerical demonstration uses a literature-constrained cytosine-level methylation matrix. These biological constraints used to construct the matrix were based on previous studies of the peloric phenotype and plant DNA methylation. Cubas et al. (1999) identified the peloric phenotype of *Linaria vulgaris* as a heritable epigenetic variant associated with methylation and transcriptional silencing of *Lcyc*, a CYCLOIDEA-like gene involved in dorsoventral floral symmetry (**Figure 1**). Ehrenhofer-Murray (2021) subsequently reported increased CG, CHG, and CHH methylation at the *Lcyc* locus in the Peloria mutant using bisulfite treatment coupled to next-generation sequencing. However, the underlying cytosine-level sequencing counts from that study are not publicly available in a form that permits direct reanalysis within the present information-theoretic framework. Consequently, we used a synthetic, literature-constrained cytosine-level dataset designed to reproduce the qualitative methylation patterns reported for the wild-type and peloric Lcyc states.

General plant methylation context was based on the known occurrence of cytosine methylation in CG, CHG, and CHH sequence contexts in plants, as described by Law and Jacobsen (2010), He et al. (2011), and related work. Methodological context for targeted bisulfite sequencing and cytosine-level methylation analysis was informed by Gruntman et al. (2008) and Masser et al. (2015). Throughout the manuscript, we therefore refer to this dataset as a synthetic, literature-constrained demonstration matrix. It is not intended to reconstruct the actual cytosine-level methylation profile of natural wild-type or peloric Lcyc alleles.

The matrix was therefore constrained to reproduce the qualitative biological pattern reported in the literature: relatively low methylation in the wild-type *Lcyc* state and increased methylation in the peloric state, particularly in non-CG contexts. The purpose of this matrix was to provide a transparent cytosine-level input dataset with which methylation capacity, entropy, divergence, context-specific contributions, and dimensionality could be calculated reproducibly.

### 2.2 Construction of the cytosine-level methylation matrix

The cytosine-level methylation matrix containing 120 potentially methylatable cytosines was constructed. The cytosines were distributed across three plant methylation contexts: 20 CG sites, 40 CHG sites, and 60 CHH sites. This distribution was chosen to represent a moderate-sized targeted bisulfite region while preserving the multicontext architecture of plant DNA methylation. The numbers of sites, their contextual distribution, and the fixed read coverage are modeling choices adopted for methodological illustration and are not presented as experimentally determined properties of the natural Lcyc locus.

For each cytosine, the matrix specifies a cytosine identifier, fragment assignment, genomic position, strand, sequence context, total read coverage, methylated read count in the wild-type state, methylated read count in the peloric state, corrected methylation probabilities, Shannon entropy (Shannon, 1948), directional Kullback-Leibler divergence (Kullback and Leibler, 1951; Cover and Thomas, 2006), symmetric KL divergence, and Jensen-Shannon divergence (Lin, 1991).

Read coverage was fixed at 50 reads per cytosine per state. This fixed-coverage design was used to isolate the behavior of the information-theoretic calculations from variation in sequencing depth. Integer methylated-read counts were selected so that the resulting context-level methylation probabilities reproduced the intended literature-constrained pattern: low methylation in wild-type plants and increased methylation in peloric plants, with the largest increases in CHG and CHH contexts.

The complete cytosine-level matrix is provided as **Supplementary Table S1**. For readability, it is divided into three context-specific tables: **Supplementary Table S1A** for CG cytosines, **Supplementary Table S1B** for CHG cytosines, and **Supplementary Table S1C** for CHH cytosines. The complete workflow, from cytosine-level counts to corrected methylation probabilities, site-wise information quantities, context-specific summaries, and dimensionality analysis, is summarized in **Figure 2**.

**Figure 2:**
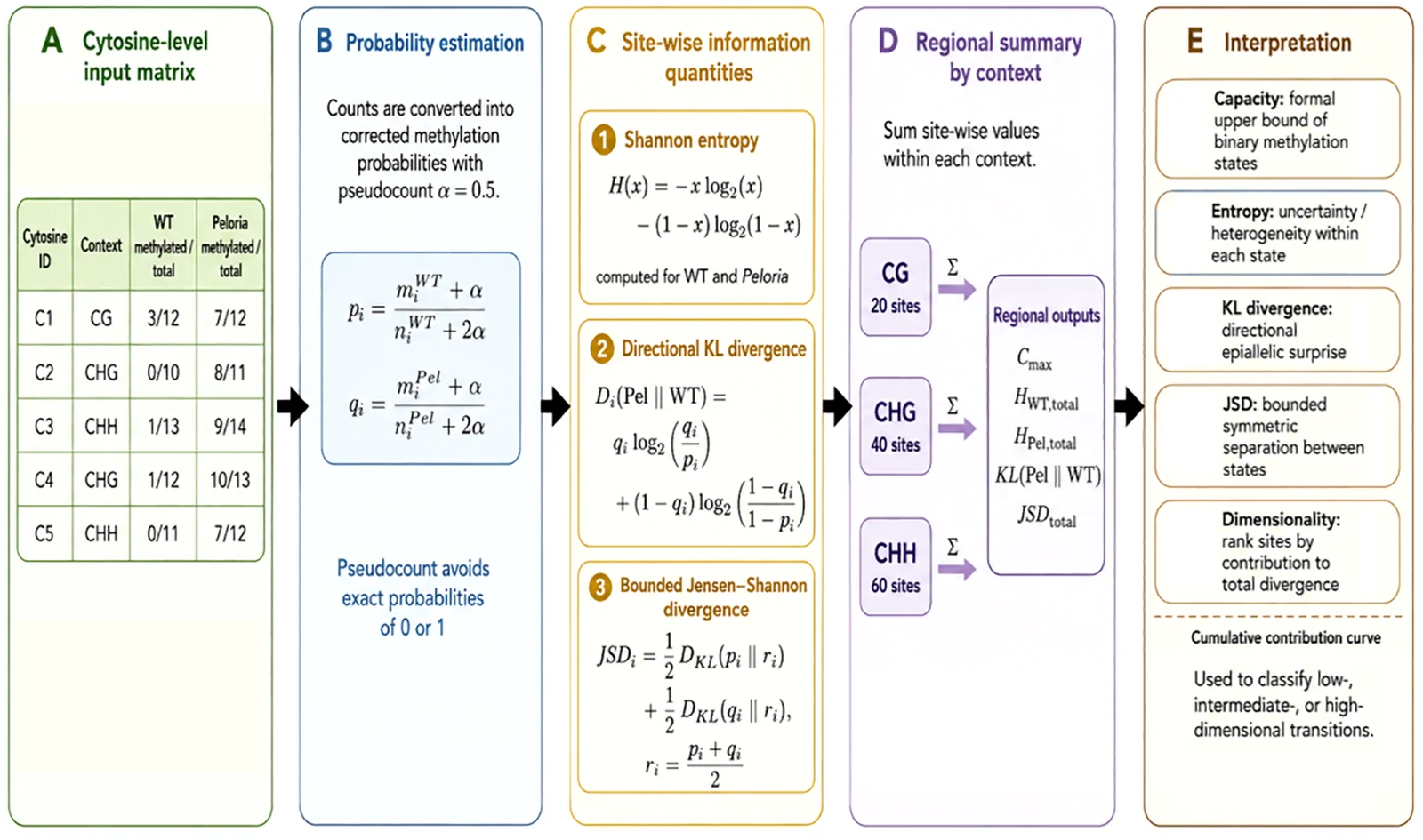
Information-theoretic workflow for the Lcyc cytosine-level matrix. Schematic representation of the computational framework used to convert cytosine-level methylation counts into information-theoretic quantities. **(A)** The input consists of a literature-constrained methylation matrix containing 120 cytosines distributed across CG, CHG, and CHH sequence contexts. For each cytosine, methylated and total read counts are specified for wild-type and peloric states. **(B)** Corrected methylation probabilities are then estimated using a pseudocount correction with (α = 0.5). **(C)** These probabilities are used to calculate site-specific Shannon entropy, directional Kullback-Leibler divergence, and summed site-wise Jensen–Shannon divergence. **(D)** Site-level values are then summed by methylation context and across the full assayed Lcyc region to obtain estimates of methylation entropy, epiallelic divergence, and context-specific contributions. **(E)** The same matrix also allows cytosines to be ranked by their contribution to total divergence, providing an operational estimate of the dimensionality of the epigenetic transition.

### 2.3 Probability estimation and pseudocount correction

For each cytosine i, methylation probabilities were estimated separately for the wild-type and peloric states. Since entropy and divergence calculations contain logarithmic terms, exact probabilities of 0 or 1 can generate undefined or unstable values. To avoid this problem, a pseudocount correction was applied with α=0.5.

The corrected wild-type (WT) methylation probability was calculated as:


$$p_{i}=\frac{m_{i}^{WT}+\alpha }{n_{i}^{WT}+2\alpha }$$

and the corrected peloric methylation probability was calculated as:


$$q_{i}=\frac{m_{i}^{Pel}+\alpha }{n_{i}^{Pel}+2\alpha }$$

where $m_{i}^{WT}$ and $m_{i}^{Pel}$ are the methylated read counts for cytosine *i* in the wild-type and peloric states, respectively, $n_{i}^{WT}$ and $n_{i}^{Pel}$ are the corresponding total read coverages, and α=0.5. All downstream entropy and divergence calculations were performed using these corrected probabilities.

### 2.4 Maximum binary capacity

The maximum binary capacity of the assayed region was defined as the number of potentially methylatable cytosines analyzed. Under the simplifying assumption that each cytosine can occupy one of two possible states, methylated or unmethylated, a region containing N cytosines has a maximum binary capacity of N bits.

The total number of cytosines was decomposed by sequence context as:


$$N=N_{CG}+N_{CHG}+N_{CHH}$$

and the maximum binary capacity was defined as:


$$C_{\text{max}}=N\text{ bits}=N_{CG}+N_{CHG}+N_{CHH}\text{ bits}$$

In the framework, the region contains 120 cytosines. Therefore, the theoretical maximum capacity of the *Lcyc* region is $C_{\max}=120$ bits. This value should be interpreted as a formal upper bound.

### 2.5 Shannon entropy of methylation states

Each cytosine was modeled as a Bernoulli methylation variable (MacKay, 2003). For a cytosine with corrected methylation probability *x*, the Shannon entropy was calculated as:


$$H\left(x\right)=-x\log_{2}\left(x\right)-\left(1-x\right)\log_{2}\left(1-x\right)$$

The site-specific entropy values for the wild-type and peloric states were therefore:


$$H_{i}^{WT}=-p_{i}\log_{2}\left(p_{i}\right)-\left(1-p_{i}\right)\log_{2}\left(1-p_{i}\right)$$

$$H_{i}^{Pel}=-q_{i}\log_{2}\left(q_{i}\right)-\left(1-q_{i}\right)\log_{2}\left(1-q_{i}\right)$$

Total methylation entropy across the assayed region was calculated by summing site-specific entropy values across all cytosines:


$$H_{WT,total}=\sum_{i=1}^{N}H_{i}^{WT}$$

$$H_{Pel,total}=\sum_{i=1}^{N}H_{i}^{Pel}$$

This additive quantity corresponds to an independence-based approximation. In general, the true joint methylation entropy satisfies $\text{H}\left({\text{X}}_{1},\ldots ,{\text{X}}_{\text{N}}\right)\leq \sum_{\text{i}}\text{H}\left({\text{X}}_{\text{i}}\right)$, with equality only when cytosine states are statistically independent. The reported summed site-wise entropy should therefore be interpreted as an upper estimate of joint entropy under the factorized approximation.

This site-wise summation assumes independence among cytosines. The independence assumption was used to provide a transparent first-order estimate. If long-read bisulfite sequencing, single-molecule methylation profiles, or complete epihaplotype data become available, the framework could be extended to joint entropy, mutual information, redundancy, and higher-order dependency structure.

### 2.6 Directional Kullback-Leibler divergence

To estimate the information-theoretic separation between wild-type and peloric methylation states, each cytosine was modeled as a Bernoulli variable and the directional Kullback-Leibler divergence was calculated. The Bernoulli Kullback-Leibler divergence from the peloric state to the wild-type state was defined as:


$$D_{i}\left(Pel∥WT\right)=q_{i}\log_{2}\left(\frac{q_{i}}{p_{i}}\right)+\left(1-q_{i}\right)\log_{2}\left(\frac{1-q_{i}}{1-p_{i}}\right)$$

The total directional divergence across the assayed region was obtained by summing site-specific values:


$$D_{total}\left(Pel∥WT\right)=\sum_{i=1}^{N}D_{i}\left(Pel∥WT\right)$$

The reverse divergence, from wild type to Peloria, was calculated as:


$$D_{i}\left(WT∥Pel\right)=p_{i}\log_{2}\left(\frac{p_{i}}{q_{i}}\right)+\left(1-p_{i}\right)\log_{2}\left(\frac{1-p_{i}}{1-q_{i}}\right)$$

Kullback-Leibler divergence is directional and unbounded. Therefore, D(Pel∥WT) and D(WT∥Pel) need not be equal and should not be interpreted as symmetric distances. In this framework, directional KL divergence was used to quantify how surprising the peloric methylation profile would be if evaluated relative to the wild-type methylation distribution.

The summed site-specific KL divergence is exactly the KL divergence between the corresponding product-Bernoulli approximations of the two methylation profiles. It therefore quantifies marginal site-wise separation and should not be interpreted as the KL divergence between unknown biological joint methylation distributions.

### 2.7 Symmetric KL divergence and summed site-wise Jensen–Shannon divergence

As a complementary symmetric measure, a symmetric KL divergence was calculated as the average of both KL directions:


$$D_{i,sym}=\frac{1}{2}D_{i}\left(Pel∥WT\right)+\frac{1}{2}D_{i}\left(WT∥Pel\right)$$

This quantity was used for ranking cytosines in the dimensionality analysis. Because symmetric KL divergence is not bounded by 1 bit per cytosine, it was not interpreted as a bounded information capacity.

A bounded symmetric alternative was obtained using summed site-wise Jensen–Shannon divergence (JSD_Σ_). For each cytosine, the midpoint methylation probability was defined as:


$$r_{i}=\frac{1}{2}\left(p_{i}+q_{i}\right)$$

The site-specific Jensen-Shannon divergence was calculated as:


$$JSD_{i}=\frac{1}{2}D_{KL}\left(q_{i}∥r_{i}\right)+\frac{1}{2}D_{KL}\left(p_{i}∥r_{i}\right)$$

where each *D_KL_* term is the Bernoulli Kullback-Leibler divergence between the corresponding methylation probability and the midpoint probability. The summed site-wise Jensen–Shannon divergence was calculated as:


$$JSD_{total}=\sum_{i=1}^{N}JSD_{i}$$

Because each cytosine is represented as a Bernoulli variable, *JSD_i_* is bounded above by 1 bit. Therefore, for a matrix containing 120 cytosines, the total summed site-wise Jensen–Shannon divergence cannot exceed 120 bits. In the present framework, JSD_Σ_ was used as an additive symmetric summary of marginal site-wise separation between the wild-type and peloric methylation profiles.

### 2.8 Context-specific decomposition

To evaluate the contribution of different plant methylation contexts to the epiallelic transition, entropy and divergence values were decomposed by CG, CHG, and CHH context. Let *S_CG_*, *S_CHG_*, and *S_CHH_* denote the sets of cytosines belonging to the CG, CHG, and CHH contexts, respectively:


$$S_{CG}=\left\{i:\text{cytosine }i\text{ belongs to the CG context}\right\}$$

$$S_{CHG}=\left\{i:\text{cytosine }i\text{ belongs to the CHG context}\right\}$$

$$S_{CHH}=\left\{i:\text{cytosine }i\text{ belongs to the CHH context}\right\}$$

For any site-specific information quantity *X_i_*, such as Shannon entropy, directional Kullback–Leibler divergence, symmetric KL divergence, summed site-wise Jensen–Shannon divergence, or site-specific contribution to total divergence, the context-specific contributions were calculated as:


$$X_{CG}=\sum_{i\in S_{CG}}X_{i}$$

$$X_{CHG}=\sum_{i\in S_{CHG}}X_{i}$$

$$X_{CHH}=\sum_{i\in S_{CHH}}X_{i}$$

The total regional value was then obtained as:


$$X_{total}=X_{CG}+X_{CHG}+X_{CHH}$$

which is equivalent to summing the site-specific quantity over all assayed cytosines:


$$X_{total}=\sum_{i=1}^{N}X_{i}$$

This decomposition was applied to Shannon entropy, directional KL divergence, symmetric KL divergence, and summed site-wise Jensen–Shannon divergence. The purpose of this analysis was to determine whether the epiallelic separation was dominated by CG methylation, non-CG methylation, or a broader multicontext methylation architecture.

### 2.9 Dimensionality analysis

The dimensionality measure used here is therefore a site-wise operational dimensionality. It quantifies how many cytosines are required to account for a specified fraction of summed marginal divergence and should not be interpreted as the number of statistically independent epigenetic degrees of freedom. Correlated groups of cytosines may represent fewer effective dimensions than the number of contributing sites suggests.

The dimensionality of the epigenetic transition was evaluated by ranking cytosines according to their site-specific contribution to total symmetric divergence. For each cytosine, the fractional contribution to total symmetric divergence was calculated as:


$$\rho_{i}=\frac{D_{i,sym}}{\sum_{j=1}^{N}D_{j,sym}}$$

Cytosines were then ranked in descending order according to *ρ_i_*. The cumulative contribution after the first *k* ranked cytosines was calculated as:


$$R\left(k\right)=\sum_{i=1}^{k}\rho_{\left(i\right)}$$

where $\rho_{\left(i\right)}$ denotes the *i*-th largest ranked contribution. The number of cytosines required to reach a given cumulative threshold *τ* was defined as:


$$K_{\tau }=\min\left\{k:R\left(k\right)\geq \tau \right\}$$

These values were chosen as round, conventional cut-offs spanning from half to nearly all of the cumulative divergence, and carry no biological meaning in themselves; they are heuristic operational descriptors rather than fixed biological categories. **Supplementary Note S7** reports N_tau at 50%, 70%, 80%, 90%, and 95% under two ranking measures (**Supplementary Table S7A**), together with threshold-free effective numbers of contributing sites (**Supplementary Table S7B**), confirming that the intermediate-to-high dimensional classification does not depend on the particular thresholds chosen.

### 2.10 Reproducibility and supplementary data

The full matrix is provided as **Supplementary Table S1**. **Supplementary Table S1A** contains the CG-context cytosines, **Supplementary Table S1B** contains the CHG-context cytosines, and **Supplementary Table S1C** contains the CHH-context cytosines. Each table reports the cytosine identifier, fragment, position, strand, methylation context, wild-type and peloric read counts, corrected methylation probabilities, Shannon entropy values, KL divergence values, symmetric KL divergence, Jensen-Shannon divergence, and source-basis label.

All rows are labeled as literature-constrained to indicate that the matrix is a reproducible demonstration dataset constrained by the literature. All logarithms were calculated in base 2, the total read coverage was fixed at 50 reads per cytosine per state, the random seed was 4480, and the pseudocount correction used α=0.5, which avoids exact probabilities of 0 or 1:


$$p=\frac{m+0.5}{n+1}$$

Here, m represents the methylated read count at a given cytosine, and n represents the total number of reads covering that cytosine. Entropy, Kullback-Leibler divergence, symmetric divergence, and Jensen-Shannon divergence are expressed in bits.

The seed controls only the initial draw of candidate methylated-read counts, which were subsequently adjusted to the target context-level methylation fractions; the final matrix is the fixed set of integer counts reported in **Supplementary Table S1**.

To evaluate the dependence of the information-theoretic outputs on assumptions of the numerical demonstration, we performed a sensitivity analysis varying pseudocount magnitude ( α=0.10−2.00), effective read coverage (10–200 reads per cytosine and state), and the magnitude of the modeled wild-type-Peloria methylation contrast. For each scenario, total Shannon entropy, directional KL divergence, summed site-wise Jensen–Shannon divergence, and context-specific contributions were recalculated. Full procedures and numerical results are provided in **Supplementary Section S2** and **Supplementary Tables S2A**-**S2C**.

To demonstrate that the framework is directly applicable to empirical methylation data, we additionally analyzed publicly available cytosine-level BSPCR counts from the *Arabidopsis thaliana FWA* locus (Ghoshal et al., 2021; GEO GSE149840). An inactive dMQ1 control (GSM4514077) was compared with an active MQ1v sample (GSM4514078). Cytosines shared between samples and covered by at least five reads in each condition were analyzed using the same pseudocount, entropy, divergence, context-decomposition, and dimensionality procedures described above. Full details are provided in **Supplementary Section S3**.

The regional Jensen-Shannon statistic used here is the sum of conventional Bernoulli JSD values calculated independently at each cytosine (JSD_Σ = Σ_i JSD_i). Each site-specific JSD is bounded by 1 bit, whereas the additive regional statistic is bounded by N bits. JSD_Σ is therefore an additive marginal summary and should not be interpreted as a single conventional JSD between full regional joint distributions. Dimensionality is reported as site-wise divergence dimensionality. In addition to cumulative thresholds, robustness was evaluated using alternative site-ranking measures and threshold-free effective numbers of contributing sites (**Supplementary Note S7**).

## 3 Results of the synthetic demonstration

### 3.1 Shannon entropy of wild-type and peloric methylation states

Using the pseudocount-corrected cytosine-level methylation probabilities defined in the Methods section, Shannon entropy (Shannon, 1948) was calculated independently for each cytosine in the wild-type and peloric states. Site-specific entropy values were then summed across the full matrix and decomposed by methylation context.

In the synthetic, literature-constrained demonstration matrix, total methylation entropy increased from 39.85 bits in the modeled wild-type state to 101.32 bits in the modeled peloric state (**Table 1**). These values are properties of the demonstration matrix under the imposed methylation probabilities and should not be interpreted as estimates obtained from natural Lcyc samples.

**Table 1:** Summary of the synthetic, literature-constrained cytosine-level matrix used solely to demonstrate the information-theoretic framework.

Context	N sites	Mean WT methylation	Mean Peloria methylation	WT entropy (bits)	Peloria entropy (bits)	KL Peloria||WT (bits)	summed site-wise JSD (bits)
CG	20	0.246	0.563	15.41	18.85	9.49	2.10
CHG	40	0.049	0.758	10.90	30.15	109.39	18.05
CHH	60	0.038	0.658	13.54	52.33	141.43	22.16
**Total**	**120**	**0.076**	**0.675**	**39.85**	**101.32**	**260.30**	**42.31**

Cytosine-level methylation counts can be converted into methylation probabilities, Shannon entropy, Kullback-Leibler divergence, summed site-wise Jensen–Shannon divergence, and context-specific contributions in bits. The literature-constrained cytosine-level matrix assumes increased methylation in Peloria relative to wild type, with especially strong increases in CHG and CHH contexts. Values in bold correspond to the Total row, calculated across all 120 cytosines (sums for entropy and divergence; means for methylation).

The context-specific entropy values showed that the peloric state had higher entropy than the wild-type state across all three methylation contexts. In absolute terms, CHH cytosines contributed the largest amount of entropy in the peloric state, followed by CHG and CG cytosines. This pattern reflects both the larger number of CHH sites in the matrix and the elevated non-CG methylation probabilities assigned to the peloric state.

### 3.2 Informational divergence between wild-type and peloric epiallelic states

The information-theoretic separation between wild-type and peloric methylation profiles was quantified using the divergence measures defined in the Methods section. Directional Kullback-Leibler divergence (Kullback and Leibler, 1951; Cover and Thomas, 2006) was used to estimate how strongly the peloric methylation profile differs from the wild-type distribution, whereas summed site-wise Jensen–Shannon divergence was used as a symmetric measure of separation between the two epiallelic states.

In the matrix, the total directional Kullback-Leibler divergence from Peloria to wild type was 260.30 bits (**Table 1**). Most of this divergence arose from non-CG methylation. CHG cytosines contributed 109.39 bits and CHH cytosines contributed 141.43 bits, whereas CG cytosines contributed 9.49 bits. This result is consistent with previous studies showing that the peloric (*Lcyc*) epiallele is associated with increased methylation across CG, CHG, and CHH contexts, with particularly strong contributions from non-CG methylation (Cubas et al., 1999; Ehrenhofer-Murray, 2021).

Because Kullback-Leibler divergence is directional and unbounded, it was complemented with Jensen-Shannon divergence, which provides a bounded symmetric measure for Bernoulli methylation variables. In the matrix, the total summed site-wise Jensen–Shannon divergence was 42.31 bits (**Table 1**). This value is mathematically consistent with the 120-cytosine matrix, because the total summed site-wise Jensen–Shannon divergence cannot exceed 120 bits when each cytosine is modeled as a Bernoulli variable.

Both quantities (260.30 and 42.31 bits) describe the separation encoded in the illustrative matrix and are presented to demonstrate the behavior and interpretation of the proposed metrics rather than to estimate the empirical divergence of natural Lcyc epialleles.

Together, these results show that the wild-type and peloric methylation profiles are strongly distinguishable in information-theoretic terms. Directional KL divergence emphasizes the magnitude of the peloric departure from the wild-type methylation distribution, whereas summed site-wise Jensen–Shannon divergence provides a conservative symmetric estimate of epiallelic separation. These context-level patterns of methylation probability, Shannon entropy, directional KL divergence, and summed site-wise Jensen–Shannon divergence are summarized graphically in **Figure 3**.

**Figure 3:**
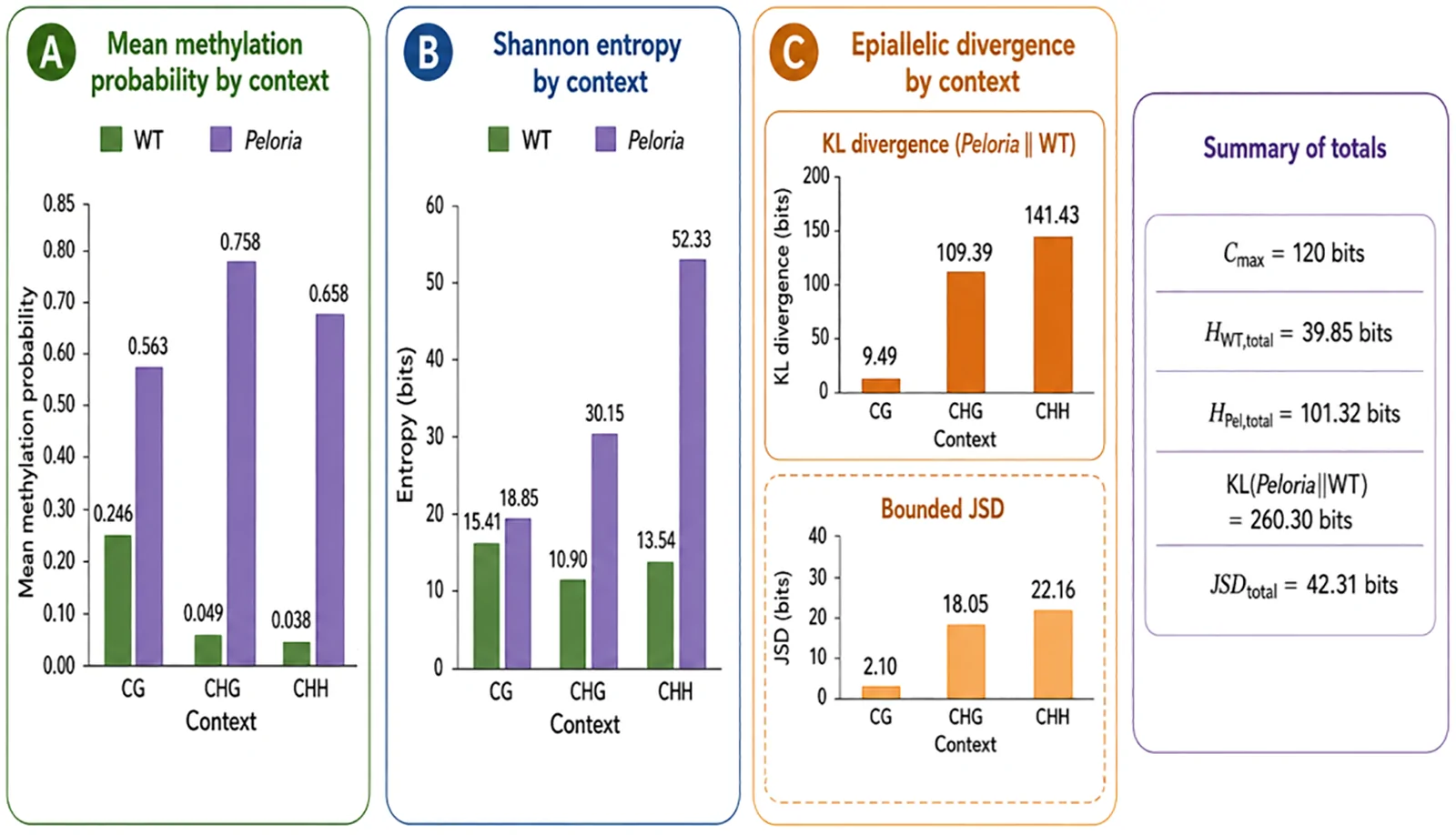
Synthetic, literature-constrained demonstration of *Lcyc* methylation. Variations of cytosine-level methylation at the *Lcyc* locus under biologically constrained assumptions. Panel **(A)** shows mean methylation probability by context in wild-type and peloric states. The framework also assumes lower methylation in wild-type plants and increased methylation in peloric plants, particularly in non-CG contexts. Panel **(B)** shows Shannon entropy by context for wild-type and peloric methylation states. Panel **(C)** shows epiallelic divergence by context, calculated as directional Kullback–Leibler divergence from Peloria to wild type and summed site-wise Jensen–Shannon divergence. The values shown are illustrative outputs of the synthetic matrix and not measurements from natural Lcyc samples.

### 3.3 Context-specific decomposition of epiallelic divergence

Because plant DNA methylation occurs in CG, CHG, and CHH contexts, the total entropy and divergence values were decomposed by sequence context (Ehrenhofer-Murray, 2021). This decomposition allows the framework to determine whether the epiallelic separation is dominated by CG methylation, non-CG methylation, or a combined multicontext methylation architecture.

In the matrix, non-CG contexts dominated the directional divergence between peloric and wild-type states. CHG and CHH cytosines contributed 109.39 and 141.43 bits, respectively, to D_total(Pel||WT). Together, these two contexts accounted for 250.82 of the 260.30 bits of directional KL divergence, corresponding to approximately 96.4% of the total. CG cytosines contributed only 9.49 bits (**Figure 3**).

A similar pattern was observed using summed site-wise Jensen–Shannon divergence. CHG and CHH cytosines contributed 18.05 and 22.16 bits, respectively, whereas CG cytosines contributed 2.10 bits (**Figure 3**). Together, non-CG cytosines accounted for 40.21 of the 42.31 bits of total summed site-wise Jensen–Shannon divergence, corresponding to approximately 95.0% of the total. These results illustrate how the framework can identify a transition dominated by non-CG methylation.

### 3.4 Dimensionality of the epigenetic transition

The contribution of each cytosine to total divergence was ranked to evaluate whether the epigenetic transition behaves as a low-, intermediate-, or high-dimensional change. If a small number of cytosines accounts for most of the divergence, the transition can be described as low-dimensional. If divergence is distributed across many cytosines, the transition is better described as a regional high-dimensional epigenetic state.

Using the symmetric site-specific divergence D_i,sym_, the fractional contribution of cytosine i to the total symmetric divergence is:


$$\rho_{i}=\frac{D_{i,sym}}{\sum_{j=1}^{N}D_{j,sym}}$$

The ranked cumulative contribution after the first k cytosines is then:


$$R\left(k\right)=\sum_{i=1}^{k}\rho_{\left(i\right)}$$

where ρ_(i)_ denotes the i-th largest ranked contribution. The number of cytosines required to reach 50%, 80%, or 90% of total divergence provides an operational measure of dimensionality:


$$K_{\tau }=\min\left\{k:R\left(k\right)\geq \tau \right\}$$

where τ is a chosen threshold such as 0.5, 0.8, or 0.9. In our framework, 38 sites explained 50% of the total symmetric divergence, 72 sites explained 80%, and 88 sites explained 90%. The ranked cumulative contribution curve and the context composition of the highest-impact cytosines are shown in **Figure 4**.

**Figure 4:**
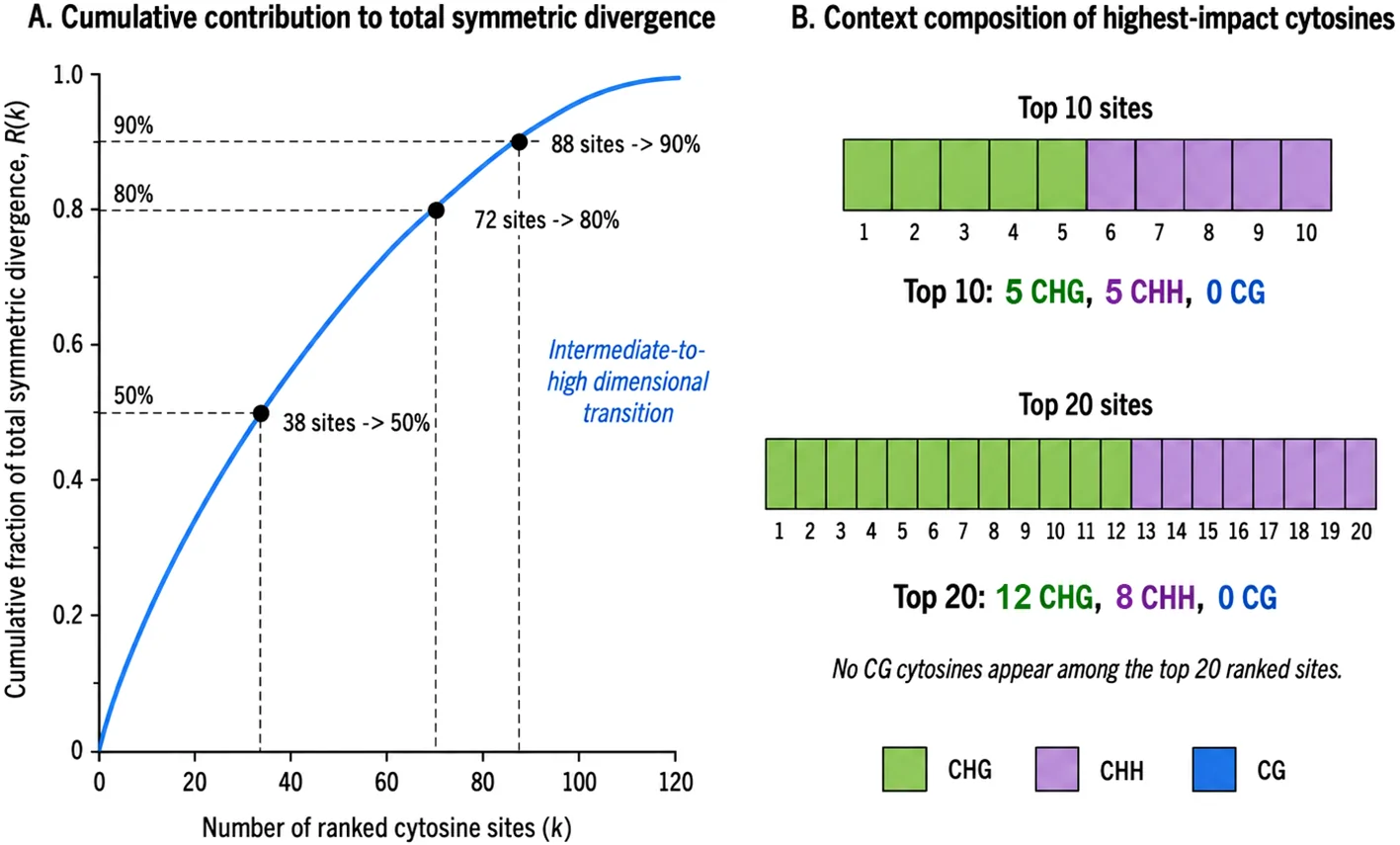
Dimensionality of the synthetic, literature-constrained epigenetic transition. Ranking of cytosine sites by their contribution to total symmetric divergence in the literature-constrained cytosine-level matrix. Panel **(A)** shows the cumulative fraction of total symmetric divergence explained by ranked cytosine sites. In the matrix, 38 sites explain 50% of total divergence, 72 sites explain 80%, and 88 sites explain 90%. Panel **(B)** summarizes the context composition of the highest-impact cytosines. Among the top 10 ranked sites, 5 belong to the CHG context and 5 to the CHH context; among the top 20 ranked sites, 12 belong to CHG and 8 to CHH. No CG cytosines appear among the top 20 ranked sites. Under the assumptions encoded in the synthetic demonstration matrix, these results illustrate an intermediate-to-high site-wise dimensionality dominated by non-CG contributions; they should not be interpreted as an empirical dimensionality estimate of the natural Lcyc epiallele.

To identify which methylation contexts contributed most strongly to the divergence, all cytosines were ranked in descending order according to their site-specific contribution to total symmetric divergence, ρ_i_. The context of the highest-ranked sites was then inspected. Among the top 10 cytosines, 5 belonged to the CHG context and 5 to the CHH context. Among the top 20 cytosines, 12 belonged to CHG and 8 to CHH. No CG cytosines appeared among these highest-ranked sites, indicating that the largest individual contributions to epiallelic separation in the literature-constrained cytosine-level matrix came from non-CG methylation. Under the assumptions encoded in the demonstration matrix, this pattern produces an intermediate-to-high-dimensional transition. This classification therefore illustrates the proposed dimensionality criterion and should not be interpreted as an empirical dimensionality estimate for the natural epiallele. The conceptual distinction between low-, intermediate-, and high-dimensional epigenetic transitions is illustrated in **Figure 5**.

**Figure 5:**
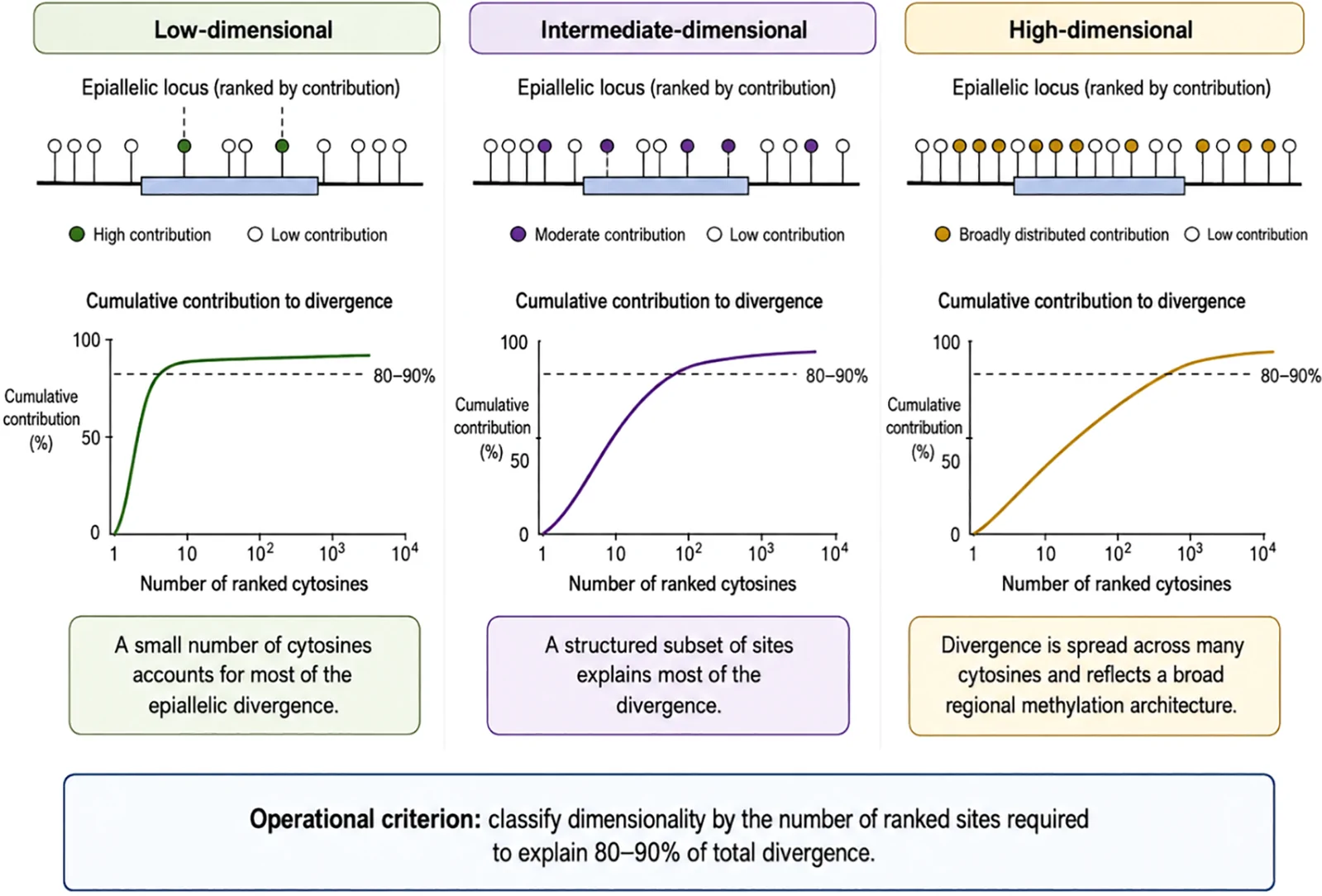
Conceptual classes of epigenetic dimensionality. Operational classification of epigenetic transitions according to the distribution of site-specific contributions to total epiallelic divergence. In a low-dimensional transition, a small number of cytosines accounts for most of the divergence between epiallelic states. In an intermediate-dimensional transition, a structured subset of sites explains most of the divergence. In a high-dimensional transition, divergence is broadly distributed across many cytosines and reflects a regional methylation architecture. Dimensionality can be quantified by ranking cytosines according to their contribution to total divergence and determining how many sites are required to explain 80–90% of the total. These thresholds are heuristic and should be refined using empirical methylation data, sequencing depth, biological replication, and locus-specific methylation architecture.

Sensitivity analysis showed that absolute information-theoretic values were influenced by probability regularization and effective read coverage, with directional KL divergence showing greater parameter sensitivity than summed site-wise Jensen–Shannon divergence. Nevertheless, the main context-specific pattern of the demonstration was highly stable: across the tested pseudocount and coverage ranges, non-CG cytosines consistently accounted for approximately 95-97% of total divergence. Moreover, experimentally motivated scaling of the modeled wild-type-Peloria methylation contrast produced monotonic changes in both KL divergence and JSD in the expected direction (**Supplementary Section S2**; **Supplementary Tables S2A**-**S2C**).

As an independent empirical demonstration of applicability, we applied the same information-theoretic framework to publicly available cytosine-level bisulfite-sequencing data from the Arabidopsis thaliana FWA locus. In contrast to the synthetic Lcyc demonstration, this analysis used experimentally observed methylated and unmethylated read counts with variable site-specific coverage. The framework could be applied without methodological modification to calculate summed site-wise entropy, directional KL divergence, summed site-wise Jensen–Shannon divergence, context-specific contributions, and site-wise dimensionality. Full methodological details and cytosine-level results are provided in **Supplementary Section S3**. Notably, the empirical FWA dataset produced a more concentrated and predominantly CG-associated divergence pattern than the synthetic Lcyc demonstration, illustrating that the framework can resolve qualitatively different methylation architectures.

The synthetic 120-site matrix yielded a summed site-wise JSD of 42.31 bits. This value represents the sum of the bounded Bernoulli JSD contributions across cytosines and is not a conventional joint-distribution JSD. Dimensionality was robust to alternative operational definitions: symmetric-KL and site-wise-JSD rankings both required many sites to explain the cumulative divergence, and threshold-free effective-site measures likewise indicated broadly distributed contributions (**Supplementary Note S7**).

## 4 Discussion

The peloric form of Linaria vulgaris occupies a special place in the history of developmental biology because it connects a classical morphological variant, first described in the eighteenth century, with a heritable epigenetic alteration at a defined regulatory locus. The central question addressed in this work is how such an epiallelic transition can be expressed quantitatively in units of information. By treating cytosine methylation states as binary variables, the framework developed here provides a way to describe the peloric *Lcyc* epiallele in terms of maximum capacity, methylation entropy, epiallelic divergence, and dimensionality.

A major conceptual distinction of the framework is the separation between methylation level and information-theoretic quantities. Increased methylation does not necessarily imply increased entropy. A fully unmethylated site and a fully methylated site both have low entropy because their states are predictable. Entropy is maximal when methylated and unmethylated states are equally probable. Therefore, methylation entropy measures uncertainty or heterogeneity, not simply methylation abundance. This distinction is important for interpreting epigenetic states such as the peloric *Lcyc* epiallele, where the biological phenotype is associated with increased methylation but the informational structure depends on the distribution of methylation probabilities across cytosines, sequence contexts, molecules, tissues, and individuals.

The synthetic, biologically constrained demonstration illustrates how the proposed framework behaves under a plausible methylation architecture. The resulting numerical values characterize the constructed matrix rather than the natural Lcyc epiallele. In our literature-constrained cytosine-level matrix, the results show that the total methylation entropy increases from 39.85 bits in the wild-type state to 101.32 bits in the peloric state. The divergence analysis provides a complementary perspective. Whereas entropy describes uncertainty within a given state, epiallelic divergence describes how distinguishable two states are from one another. In the case study, the total Kullback-Leibler divergence from Peloria to wild type is 260.30 bits, and the summed site-wise Jensen-Shannon divergence is 42.31 bits. They represent the information-theoretic distinguishability of the peloric and wild-type methylation distributions under the assumptions of our framework.

Throughout this study, information is used in the Shannon-theoretic statistical sense. Entropy and divergence quantify uncertainty and statistical distinguishability of methylation-state distributions; they do not measure functional biological information or causal contribution to floral morphology. Functional interpretation requires independent experimental evidence linking methylation configurations to gene regulation, chromatin state, developmental context, and phenotype.

A biologically motivated feature built into the demonstration matrix is the stronger contribution of non-CG methylation. Consequently, the predominance of CHG and CHH divergence in the numerical results should be regarded as an illustration of how the framework resolves context-specific contributions, rather than as an independent empirical discovery about Lcyc. In plants, CG, CHG, and CHH methylation are maintained by partially distinct molecular pathways and may differ in stability, inheritance, and regulatory significance. Therefore, decomposing entropy and divergence by sequence context is not merely a technical convenience; it is biologically essential for interpreting the regulatory architecture of the epiallele.

The dimensionality analysis adds another layer to the interpretation. If most of the epiallelic divergence were concentrated in a very small number of cytosines, the peloric transition could be described as a low-dimensional epigenetic switch. Conversely, if divergence is distributed across many cytosines, the epiallele is better understood as a regional chromatin-level state. In the case study, 38 cytosines explain 50% of the total symmetric divergence, 72 cytosines explain 80%, and 88 cytosines explain 90%. This pattern suggests an intermediate-to-high-dimensional transition in which many non-CG sites contribute to the separation between epiallelic states.

The framework also clarifies what kind of data is required for an empirical estimate. Context-averaged methylation values are useful for describing broad biological trends, but they are insufficient for calculating site-specific entropy, divergence, and dimensionality. The essential input is a cytosine-level methylation matrix containing, for each assayed cytosine, its position, sequence context, methylated read count, and total read coverage in wild-type and peloric samples.

Several limitations should be emphasized. First, the numerical results reported here are derived from a synthetic, literature-constrained demonstration, not from empirical cytosine-level counts at the natural Lcyc locus. The sensitivity analysis further indicates that the absolute magnitudes of the information-theoretic quantities are not independent of analytical and sampling assumptions. Directional KL divergence is particularly sensitive to pseudocount choice and effective sequencing depth when site probabilities approach 0 or 1, whereas summed site-wise Jensen–Shannon divergence is comparatively less affected. Importantly, this numerical sensitivity did not alter the principal structural result of the demonstration: non-CG methylation remained responsible for approximately 95-97% of regional divergence across the tested parameter ranges. Empirical applications should therefore report sequencing depth and probability-regularization procedures explicitly and should preferably evaluate sensitivity to these choices rather than interpreting a single bit value in isolation. Second, the current model treats cytosines as independent Bernoulli variables. This assumption allows a transparent first-order estimate, but real methylation patterns may show correlations among neighboring cytosines, strand-specific effects, cell-type heterogeneity, allele-specific differences, and epihaplotype structure. If long-read bisulfite sequencing or complete methylation haplotypes become available, the framework could be extended from site-wise entropy to joint entropy, mutual information, redundancy, and higher-order dependency structure. Third, the framework quantifies distinguishability between methylation states, not causality. A cytosine that contributes strongly to divergence is not necessarily a causal determinant of floral morphology. Conversely, a cytosine with low marginal divergence might participate in a regulatory pattern through interactions with other sites.

Although Ehrenhofer-Murray (2021) generated targeted bisulfite-sequencing data for the Lcyc locus, the underlying cytosine-level count data required for direct application of the present framework were not publicly available for reanalysis. This lack of accessible count-level data motivated the use of the synthetic, literature-constrained demonstration matrix.

The distinction between statistical divergence and biological causality is central to the interpretation of this work. The peloric phenotype should not be described as being caused by a precise number of methylation bits. Developmental outcomes emerge from regulatory networks, chromatin states, transcriptional control, tissue context, and developmental timing. The information-theoretic quantities proposed here describe the molecular separation between epiallelic states, not the full causal pathway from methylation to floral morphology. Nevertheless, such quantities are useful because they provide a rigorous language for comparing epigenetic states and for asking how distributed molecular modifications relate to visible developmental transitions.

This framework may have applications beyond *Linaria vulgaris*. Many plant phenotypes are associated with heritable or metastable epigenetic variation, including changes in floral morphology, transposon activity, stress response, developmental timing, and gene expression. In each case, cytosine-level methylation data could be converted into entropy, divergence, and dimensionality profiles. This would make it possible to compare epialleles not only by whether they are methylated, but by how much information separates their regulatory states, whether that information is concentrated or distributed, and which methylation contexts contribute most strongly to the distinction.

The independent FWA analysis presented in **Supplementary Section S3** provides an empirical proof of applicability of this procedure. Using publicly available cytosine-level methylated and unmethylated read counts with heterogeneous coverage, the same equations could be applied directly without modification, demonstrating that the framework is not intrinsically dependent on the fixed-coverage or predefined-probability assumptions of the synthetic Lcyc matrix. This FWA analysis is not intended as a biological surrogate for the peloric Lcyc epiallele, but rather as an independent experimental demonstration that the general information-theoretic procedure can be applied to real methylation-count data.

In conclusion, the peloric Lcyc epiallele provides a biologically motivated case study for developing an information-theoretic framework linking molecular epigenetics and developmental morphology. The present study does not provide an empirical estimate of the Shannon-theoretic methylation-state quantities of the natural epiallele; rather, it demonstrates, using a synthetic literature-constrained matrix, how cytosine-level methylation counts can be transformed into measures of capacity, entropy, divergence, and dimensionality. Once experimental count-level data become available, the same framework can be applied directly to natural wild-type and peloric Lcyc epialleles.

## Data Availability

The original contributions presented in the study are included in the article/**Supplementary Material**. Further inquiries can be directed to the corresponding author.
