# Supplementary material for: An Information-theoretic framework for the peloric Lcyc epiallele in Linaria vulgaris: a synthetic demonstration
Source: Front Plant Sci. 2026 Oct 16;17:1915131. doi: 10.3389/fpls.2026.1915131 (PMC13719474; doi:10.3389/fpls.2026.1915131)
Supplement: Supplementary file 1 [file DataSheet1.pdf]

Supplementary material

Supplementary Table S1. Literature-constrained cytosine-level methylation matrix for the *Lcyc* epiallele information framework.

This supplementary table reports a literature-constrained cytosine-level matrix used to demonstrate the information-theoretic framework. Methylation probabilities were estimated with a pseudocount $\alpha = 0.5$: $p = (m + 0.5)/(n + 1)$. Entropy, Kullback-Leibler divergence, symmetric divergence, and Jensen-Shannon divergence are expressed in bits. Decimal values are rounded to four digits for display. Column abbreviations: WT n and Pel n = total read counts; WT m and Pel m = methylated read counts; p WT and q Pel = pseudocount-corrected methylation probabilities; H = Shannon entropy; KL = Bernoulli Kullback-Leibler divergence; Dsym = average of both KL directions; JSD = bounded Bernoulli Jensen-Shannon divergence.

S1A. CG-context cytosines (n = 20)

Cytosine ID	Fragment	Position bp	Strand	Context	Tri-context	WT n	WT m	Pel n	Pel m	p WT	q Pel	H WT	H Pel	KL Pel||WT	KL WT||Pel	Dsym	JSD
Lcyc_syn_C001	Frag 1	105	-	CG	CG	50	9	50	28	0.1863	0.5588	0.6936	0.9900	0.4961	0.4234	0.4598	0.1108
Lcyc_syn_C002	Frag 1	114	-	CG	CG	50	17	50	18	0.3431	0.3627	0.9278	0.9449	0.0012	0.0012	0.0012	0.0003
Lcyc_syn_C003	Frag 1	119	+	CG	CG	50	19	50	28	0.3824	0.5588	0.9597	0.9900	0.0918	0.0905	0.0911	0.0227
Lcyc_syn_C004	Frag 1	123	+	CG	CG	50	17	50	31	0.3431	0.6176	0.9278	0.9597	0.2253	0.2218	0.2235	0.0552
Lcyc_syn_C005	Frag 1	130	-	CG	CG	50	9	50	42	0.1863	0.8333	0.6936	0.6500	1.4200	1.4588	1.4394	0.3279
Lcyc_syn_C006	Frag 1	137	+	CG	CG	50	21	50	20	0.4216	0.4020	0.9822	0.9721	0.0011	0.0011	0.0011	0.0003
Lcyc_syn_C007	Frag 1	148	+	CG	CG	50	7	50	20	0.1471	0.4020	0.6024	0.9721	0.2768	0.2235	0.2502	0.0606
Lcyc_syn_C008	Frag 1	150	-	CG	CG	50	12	50	33	0.2451	0.6569	0.8034	0.9278	0.5439	0.5101	0.5270	0.1275
Lcyc_syn_C009	Frag 1	153	-	CG	CG	50	10	50	20	0.2059	0.4020	0.7335	0.9721	0.1433	0.1262	0.1347	0.0332
Lcyc_syn_C010	Frag 1	158	-	CG	CG	50	12	50	21	0.2451	0.4216	0.8034	0.9822	0.1076	0.0982	0.1029	0.0255
Lcyc_syn_C011	Frag 1	164	-	CG	CG	50	12	50	29	0.2451	0.5784	0.8034	0.9822	0.3622	0.3309	0.3466	0.0846
Lcyc_syn_C012	Frag 1	174	-	CG	CG	50	8	50	35	0.1667	0.6961	0.6500	0.8860	0.9932	0.8689	0.9311	0.2183
Lcyc_syn_C013	Frag 1	181	-	CG	CG	50	20	50	32	0.4020	0.6373	0.9721	0.9449	0.1620	0.1641	0.1631	0.0404
Lcyc_syn_C014	Frag 1	189	+	CG	CG	50	17	50	26	0.3431	0.5196	0.9278	0.9989	0.0942	0.0911	0.0926	0.0230
Lcyc_syn_C015	Frag 1	191	-	CG	CG	50	6	50	32	0.1275	0.6373	0.5504	0.9449	1.0203	0.8090	0.9146	0.2120
Lcyc_syn_C016	Frag 1	196	-	CG	CG	50	5	50	35	0.1078	0.6961	0.4934	0.8860	1.4005	1.0959	1.2482	0.2824
Lcyc_syn_C017	Frag 1	206	-	CG	CG	50	11	50	32	0.2255	0.6373	0.7701	0.9449	0.5582	0.5096	0.5339	0.1289
Lcyc_syn_C018	Frag 1	213	+	CG	CG	50	7	50	34	0.1471	0.6765	0.6024	0.9082	1.0369	0.8691	0.9530	0.2221
Lcyc_syn_C019	Frag 1	222	+	CG	CG	50	8	50	26	0.1667	0.5196	0.6500	0.9989	0.4706	0.3888	0.4297	0.1033
Lcyc_syn_C020	Frag 1	231	-	CG	CG	50	14	50	22	0.2843	0.4412	0.8613	0.9900	0.0802	0.0752	0.0777	0.0193

S1B. CHG-context cytosines (n = 40)

Cytosine ID	Fragment	Position bp	Strand	Context	Tri-context	WT n	WT m	Pel n	Pel m	p WT	q Pel	H WT	H Pel	KL Pel||WT	KL WT||Pel	Dsym	JSD
Lcyc_syn_C021	Frag 2	237	-	CHG	CCG	50	2	50	39	0.0490	0.7745	0.2822	0.7701	2.6158	1.7794	2.1976	0.4513
Lcyc_syn_C022	Frag 2	242	-	CHG	CTG	50	3	50	31	0.0686	0.6176	0.3608	0.9597	1.4668	0.9788	1.2228	0.2676
Lcyc_syn_C023	Frag 2	244	-	CHG	CCG	50	3	50	39	0.0686	0.7745	0.3608	0.7701	2.2466	1.6659	1.9563	0.4168
Lcyc_syn_C024	Frag 2	255	+	CHG	CCG	50	1	50	39	0.0294	0.7745	0.1914	0.7701	3.1799	1.9051	2.5425	0.4913
Lcyc_syn_C025	Frag 2	262	+	CHG	CTG	50	1	50	37	0.0294	0.7353	0.1914	0.8338	2.9184	1.6828	2.3006	0.4471
Lcyc_syn_C026	Frag 2	264	-	CHG	CAG	50	2	50	43	0.0490	0.8529	0.2822	0.6024	3.1190	2.3590	2.7390	0.5507
Lcyc_syn_C027	Frag 2	274	-	CHG	CCG	50	2	50	37	0.0490	0.7353	0.2822	0.8338	2.3843	1.5631	1.9737	0.4082
Lcyc_syn_C028	Frag 2	284	-	CHG	CAG	50	1	50	38	0.0294	0.7549	0.1914	0.8034	3.0477	1.7894	2.4185	0.4688
Lcyc_syn_C029	Frag 2	290	-	CHG	CTG	50	2	50	26	0.0490	0.5196	0.2822	0.9989	1.2965	0.7699	1.0332	0.2207
Lcyc_syn_C030	Frag 2	294	+	CHG	CCG	50	3	50	44	0.0686	0.8725	0.3608	0.5504	2.8351	2.4207	2.6279	0.5419
Lcyc_syn_C031	Frag 2	303	-	CHG	CTG	50	1	50	28	0.0294	0.5588	0.1914	0.9900	1.8720	0.9791	1.4256	0.2833
Lcyc_syn_C032	Frag 2	312	+	CHG	CCG	50	0	50	40	0.0098	0.7941	0.0795	0.7335	4.5681	2.1815	3.3748	0.5656
Lcyc_syn_C033	Frag 2	323	-	CHG	CTG	50	2	50	37	0.0490	0.7353	0.2822	0.8338	2.3843	1.5631	1.9737	0.4082
Lcyc_syn_C034	Frag 2	329	-	CHG	CCG	50	3	50	37	0.0686	0.7353	0.3608	0.8338	2.0353	1.4556	1.7455	0.3748
Lcyc_syn_C035	Frag 2	337	-	CHG	CCG	50	0	50	41	0.0098	0.8137	0.0795	0.6936	4.7386	2.3242	3.5314	0.5909
Lcyc_syn_C036	Frag 2	344	-	CHG	CCG	50	2	50	35	0.0490	0.6961	0.2822	0.8860	2.1643	1.3774	1.7708	0.3685
Lcyc_syn_C037	Frag 2	352	+	CHG	CTG	50	2	50	39	0.0490	0.7745	0.2822	0.7701	2.6158	1.7794	2.1976	0.4513
Lcyc_syn_C038	Frag 2	362	+	CHG	CCG	50	3	50	42	0.0686	0.8333	0.3608	0.6500	2.5880	2.0648	2.3264	0.4877
Lcyc_syn_C039	Frag 2	372	+	CHG	CTG	50	4	50	42	0.0882	0.8333	0.4306	0.6500	2.2909	1.9495	2.1202	0.4553
Lcyc_syn_C040	Frag 2	379	+	CHG	CAG	50	6	50	38	0.1275	0.7549	0.5504	0.8034	1.4884	1.2713	1.3798	0.3131
Lcyc_syn_C041	Frag 2	384	+	CHG	CAG	50	1	50	36	0.0294	0.7157	0.1914	0.8613	2.7920	1.5838	2.1879	0.4263
Lcyc_syn_C042	Frag 2	388	+	CHG	CAG	50	1	50	40	0.0294	0.7941	0.1914	0.7335	3.3154	2.0314	2.6734	0.5149
Lcyc_syn_C043	Frag 2	394	+	CHG	CCG	50	2	50	40	0.0490	0.7941	0.2822	0.7335	2.7362	1.9024	2.3193	0.4743
Lcyc_syn_C044	Frag 2	403	+	CHG	CAG	50	1	50	28	0.0294	0.5588	0.1914	0.9900	1.8720	0.9791	1.4256	0.2833
Lcyc_syn_C045	Frag 2	409	-	CHG	CTG	50	1	50	37	0.0294	0.7353	0.1914	0.8338	2.9184	1.6828	2.3006	0.4471
Lcyc_syn_C046	Frag 2	418	-	CHG	CTG	50	2	50	42	0.0490	0.8333	0.2822	0.6500	2.9875	2.1889	2.5882	0.5239

Cytosine ID	Fragment	Position bp	Strand	Context	Tri-context	WT n	WT m	Pel n	Pel m	p WT	q Pel	H WT	H Pel	KL Pel||WT	KL WT||Pel	Dsym	JSD
Lcyc_syn_C047	Frag 2	427	-	CHG	CTG	50	0	50	37	0.0098	0.7353	0.0795	0.8338	4.0762	1.8236	2.9499	0.4960
Lcyc_syn_C048	Frag 2	433	+	CHG	CTG	50	2	50	45	0.0490	0.8922	0.2822	0.4934	3.3958	2.7813	3.0886	0.6097
Lcyc_syn_C049	Frag 2	438	+	CHG	CTG	50	1	50	36	0.0294	0.7157	0.1914	0.8613	2.7920	1.5838	2.1879	0.4263
Lcyc_syn_C050	Frag 2	447	+	CHG	CTG	50	2	50	45	0.0490	0.8922	0.2822	0.4934	3.3958	2.7813	3.0886	0.6097
Lcyc_syn_C051	Frag 2	456	+	CHG	CAG	50	3	50	40	0.0686	0.7941	0.3608	0.7335	2.3569	1.7857	2.0713	0.4392
Lcyc_syn_C052	Frag 2	464	-	CHG	CTG	50	2	50	45	0.0490	0.8922	0.2822	0.4934	3.3958	2.7813	3.0886	0.6097
Lcyc_syn_C053	Frag 2	475	-	CHG	CAG	50	2	50	43	0.0490	0.8529	0.2822	0.6024	3.1190	2.3590	2.7390	0.5507
Lcyc_syn_C054	Frag 2	479	-	CHG	CCG	50	1	50	50	0.0294	0.9902	0.1914	0.0795	4.9585	6.2852	5.6218	0.8643
Lcyc_syn_C055	Frag 2	487	-	CHG	CAG	50	2	50	45	0.0490	0.8922	0.2822	0.4934	3.3958	2.7813	3.0886	0.6097
Lcyc_syn_C056	Frag 2	489	-	CHG	CAG	50	3	50	29	0.0686	0.5784	0.3608	0.9822	1.2967	0.8541	1.0754	0.2367
Lcyc_syn_C057	Frag 2	492	-	CHG	CAG	50	5	50	34	0.1078	0.6765	0.4934	0.9082	1.3186	1.0199	1.1692	0.2654
Lcyc_syn_C058	Frag 2	499	-	CHG	CCG	50	3	50	27	0.0686	0.5392	0.3608	0.9956	1.1358	0.7415	0.9387	0.2079
Lcyc_syn_C059	Frag 2	507	-	CHG	CAG	50	3	50	37	0.0686	0.7353	0.3608	0.8338	2.0353	1.4556	1.7455	0.3748
Lcyc_syn_C060	Frag 2	509	+	CHG	CAG	50	0	50	38	0.0098	0.7549	0.0795	0.8034	4.2371	1.9332	3.0851	0.5182

S1C. CHH-context cytosines (n = 60)

Cytosine ID	Fragment	Position bp	Strand	Context	Tri-context	WT n	WT m	Pel n	Pel m	p WT	q Pel	H WT	H Pel	KL Pel||WT	KL WT||Pel	Dsym	JSD
Lcyc_syn_C061	Frag 3	515	+	CHH	CAC	50	1	50	32	0.0294	0.6373	0.1914	0.9449	2.3127	1.2476	1.7802	0.3501
Lcyc_syn_C062	Frag 3	526	+	CHH	CAA	50	2	50	34	0.0490	0.6765	0.2822	0.9082	2.0583	1.2937	1.6760	0.3497
Lcyc_syn_C063	Frag 3	529	+	CHH	CTT	50	1	50	39	0.0294	0.7745	0.1914	0.7701	3.1799	1.9051	2.5425	0.4913
Lcyc_syn_C064	Frag 3	531	-	CHH	CTT	50	1	50	22	0.0294	0.4412	0.1914	0.9900	1.2785	0.6581	0.9683	0.1964
Lcyc_syn_C065	Frag 3	539	-	CHH	CCA	50	5	50	31	0.1078	0.6176	0.4934	0.9597	1.0878	0.8190	0.9534	0.2184
Lcyc_syn_C066	Frag 3	545	-	CHH	CCA	50	1	50	21	0.0294	0.4216	0.1914	0.9822	1.1874	0.6118	0.8996	0.1833
Lcyc_syn_C067	Frag 3	550	+	CHH	CAA	50	0	50	28	0.0098	0.5588	0.0795	0.9900	2.7450	1.0977	1.9214	0.3265
Lcyc_syn_C068	Frag 3	554	+	CHH	CTA	50	1	50	19	0.0294	0.3824	0.1914	0.9597	1.0121	0.5241	0.7681	0.1580
Lcyc_syn_C069	Frag 3	562	-	CHH	CCA	50	1	50	31	0.0294	0.6176	0.1914	0.9597	2.1990	1.1752	1.6871	0.3326
Lcyc_syn_C070	Frag 3	567	+	CHH	CCA	50	2	50	41	0.0490	0.8137	0.2822	0.6936	2.8600	2.0380	2.4490	0.4985
Lcyc_syn_C071	Frag 3	569	-	CHH	CTA	50	0	50	31	0.0098	0.6176	0.0795	0.9597	3.1670	1.3007	2.2339	0.3778
Lcyc_syn_C072	Frag 3	576	+	CHH	CTT	50	3	50	29	0.0686	0.5784	0.3608	0.9822	1.2967	0.8541	1.0754	0.2367
Lcyc_syn_C073	Frag 3	585	+	CHH	CAC	50	1	50	28	0.0294	0.5588	0.1914	0.9900	1.8720	0.9791	1.4256	0.2833
Lcyc_syn_C074	Frag 3	595	+	CHH	CCA	50	0	50	40	0.0098	0.7941	0.0795	0.7335	4.5681	2.1815	3.3748	0.5656
Lcyc_syn_C075	Frag 3	599	-	CHH	CCA	50	1	50	44	0.0294	0.8725	0.1914	0.5504	3.8941	2.6989	3.2965	0.6221
Lcyc_syn_C076	Frag 3	606	+	CHH	CTT	50	3	50	32	0.0686	0.6373	0.3608	0.9449	1.5553	1.0464	1.3009	0.2838
Lcyc_syn_C077	Frag 3	615	+	CHH	CAT	50	2	50	36	0.0490	0.7157	0.2822	0.8613	2.2729	1.4669	1.8699	0.3879
Lcyc_syn_C078	Frag 3	621	-	CHH	CAT	50	1	50	32	0.0294	0.6373	0.1914	0.9449	2.3127	1.2476	1.7802	0.3501
Lcyc_syn_C079	Frag 3	631	+	CHH	CTT	50	2	50	35	0.0490	0.6961	0.2822	0.8860	2.1643	1.3774	1.7708	0.3685
Lcyc_syn_C080	Frag 3	639	+	CHH	CTA	50	0	50	24	0.0098	0.4804	0.0795	0.9989	2.2139	0.8661	1.5400	0.2642
Lcyc_syn_C081	Frag 3	644	+	CHH	CAC	50	3	50	25	0.0686	0.5000	0.3608	1.0000	0.9838	0.6392	0.8115	0.1809
Lcyc_syn_C082	Frag 3	648	+	CHH	CTT	50	3	50	37	0.0686	0.7353	0.3608	0.8338	2.0353	1.4556	1.7455	0.3748
Lcyc_syn_C083	Frag 3	655	+	CHH	CAA	50	0	50	30	0.0098	0.5980	0.0795	0.9721	3.0240	1.2298	2.1269	0.3602
Lcyc_syn_C084	Frag 3	659	-	CHH	CAA	50	1	50	28	0.0294	0.5588	0.1914	0.9900	1.8720	0.9791	1.4256	0.2833
Lcyc_syn_C085	Frag 3	665	+	CHH	CAC	50	1	50	38	0.0294	0.7549	0.1914	0.8034	3.0477	1.7894	2.4185	0.4688
Lcyc_syn_C086	Frag 3	670	-	CHH	CAT	50	1	50	29	0.0294	0.5784	0.1914	0.9822	1.9787	1.0413	1.5100	0.2992
Lcyc_syn_C087	Frag 3	677	-	CHH	CAC	50	2	50	42	0.0490	0.8333	0.2822	0.6500	2.9875	2.1889	2.5882	0.5239
Lcyc_syn_C088	Frag 3	684	+	CHH	CTT	50	1	50	36	0.0294	0.7157	0.1914	0.8613	2.7920	1.5838	2.1879	0.4263
Lcyc_syn_C089	Frag 3	688	+	CHH	CTA	50	2	50	40	0.0490	0.7941	0.2822	0.7335	2.7362	1.9024	2.3193	0.4743
Lcyc_syn_C090	Frag 3	692	-	CHH	CAA	50	2	50	40	0.0490	0.7941	0.2822	0.7335	2.7362	1.9024	2.3193	0.4743
Lcyc_syn_C091	Frag 3	695	-	CHH	CTA	50	1	50	35	0.0294	0.6961	0.1914	0.8860	2.6683	1.4916	2.0800	0.4062
Lcyc_syn_C092	Frag 3	701	-	CHH	CAT	50	2	50	43	0.0490	0.8529	0.2822	0.6024	3.1190	2.3590	2.7390	0.5507
Lcyc_syn_C093	Frag 3	710	-	CHH	CTA	50	1	50	29	0.0294	0.5784	0.1914	0.9822	1.9787	1.0413	1.5100	0.2992
Lcyc_syn_C094	Frag 3	721	+	CHH	CTT	50	2	50	39	0.0490	0.7745	0.2822	0.7701	2.6158	1.7794	2.1976	0.4513
Lcyc_syn_C095	Frag 3	726	-	CHH	CTA	50	3	50	29	0.0686	0.5784	0.3608	0.9822	1.2967	0.8541	1.0754	0.2367
Lcyc_syn_C096	Frag 3	730	-	CHH	CAT	50	1	50	38	0.0294	0.7549	0.1914	0.8034	3.0477	1.7894	2.4185	0.4688
Lcyc_syn_C097	Frag 3	738	-	CHH	CAC	50	1	50	27	0.0294	0.5392	0.1914	0.9956	1.7675	0.9197	1.3436	0.2678
Lcyc_syn_C098	Frag 3	746	+	CHH	CAC	50	3	50	34	0.0686	0.6765	0.3608	0.9082	1.7396	1.1942	1.4669	0.3181
Lcyc_syn_C099	Frag 3	751	-	CHH	CCA	50	1	50	39	0.0294	0.7745	0.1914	0.7701	3.1799	1.9051	2.5425	0.4913
Lcyc_syn_C100	Frag 3	758	-	CHH	CTA	50	2	50	28	0.0490	0.5588	0.2822	0.9900	1.4732	0.8816	1.1774	0.2499
Lcyc_syn_C101	Frag 3	765	+	CHH	CAA	50	2	50	47	0.0490	0.9314	0.2822	0.3608	3.6961	3.3984	3.5473	0.6782
Lcyc_syn_C102	Frag 3	776	+	CHH	CAA	50	0	50	25	0.0098	0.5000	0.0795	1.0000	2.3433	0.9205	1.6319	0.2792
Lcyc_syn_C103	Frag 3	782	-	CHH	CTT	50	1	50	36	0.0294	0.7157	0.1914	0.8613	2.7920	1.5838	2.1879	0.4263
Lcyc_syn_C104	Frag 3	786	+	CHH	CTA	50	1	50	41	0.0294	0.8137	0.1914	0.6936	3.4542	2.1705	2.8124	0.5397
Lcyc_syn_C105	Frag 3	796	-	CHH	CAT	50	1	50	31	0.0294	0.6176	0.1914	0.9597	2.1990	1.1752	1.6871	0.3326
Lcyc_syn_C106	Frag 3	801	-	CHH	CAT	50	1	50	22	0.0294	0.4412	0.1914	0.9900	1.2785	0.6581	0.9683	0.1964
Lcyc_syn_C107	Frag 3	812	-	CHH	CTA	50	1	50	32	0.0294	0.6373	0.1914	0.9449	2.3127	1.2476	1.7802	0.3501
Lcyc_syn_C108	Frag 3	815	-	CHH	CAA	50	3	50	35	0.0686	0.6961	0.3608	0.8860	1.8355	1.2754	1.5555	0.3363
Lcyc_syn_C109	Frag 3	818	-	CHH	CAT	50	1	50	26	0.0294	0.5196	0.1914	0.9989	1.6653	0.8630	1.2641	0.2527
Lcyc_syn_C110	Frag 3	825	-	CHH	CAC	50	1	50	34	0.0294	0.6765	0.1914	0.9082	2.5473	1.4053	1.9763	0.3869
Lcyc_syn_C111	Frag 3	830	+	CHH	CAT	50	2	50	46	0.0490	0.9118	0.2822	0.4306	3.5425	3.0551	3.2988	0.6425
Lcyc_syn_C112	Frag 3	840	-	CHH	CAT	50	2	50	31	0.0490	0.6176	0.2822	0.9597	1.7551	1.0709	1.4130	0.2973
Lcyc_syn_C113	Frag 3	842	+	CHH	CAC	50	3	50	31	0.0686	0.6176	0.3608	0.9597	1.4668	0.9788	1.2228	0.2676
Lcyc_syn_C114	Frag 3	852	+	CHH	CTA	50	1	50	29	0.0294	0.5784	0.1914	0.9822	1.9787	1.0413	1.5100	0.2992
Lcyc_syn_C115	Frag 3	854	+	CHH	CTA	50	1	50	27	0.0294	0.5392	0.1914	0.9956	1.7675	0.9197	1.3436	0.2678
Lcyc_syn_C116	Frag 3	857	+	CHH	CAT	50	0	50	27	0.0098	0.5392	0.0795	0.9956	2.6089	1.0361	1.8225	0.3103
Lcyc_syn_C117	Frag 3	862	-	CHH	CTA	50	1	50	43	0.0294	0.8529	0.1914	0.6024	3.7432	2.4995	3.1214	0.5931
Lcyc_syn_C118	Frag 3	866	+	CHH	CTT	50	1	50	34	0.0294	0.6765	0.1914	0.9082	2.5473	1.4053	1.9763	0.3869

Cytosine ID	Fragment	Position bp	Strand	Context	Tri-context	WT n	WT m	Pel n	Pel m	p WT	q Pel	H WT	H Pel	KL Pel‖WT	KL WT‖Pel	Dsym	JSD
Lcyc_syn_C119	Frag 3	872	-	CHH	CCA	50	1	50	42	0.0294	0.8333	0.1914	0.6500	3.5967	2.3252	2.9610	0.5656
Lcyc_syn_C120	Frag 3	874	+	CHH	CTA	50	1	50	29	0.0294	0.5784	0.1914	0.9822	1.9787	1.0413	1.5100	0.2992

Supplementary Section S2. Sensitivity analysis of the information-theoretic framework

S2.1. Rationale and analysis design

The numerical demonstration presented in the main manuscript is based on a synthetic, literature-constrained cytosine-level methylation matrix containing 120 potentially methylatable cytosines. In the reference analysis, read coverage was fixed at 50 reads per cytosine and per state, methylation probabilities were regularized with a pseudocount of $\alpha = 0.5$, and the resulting site-specific probabilities were used to calculate Shannon entropy, directional Kullback-Leibler (KL) divergence, and summed site-wise Jensen–Shannon divergence (JSD). Because these choices may influence the absolute magnitude of information-theoretic quantities, we evaluated the sensitivity of the framework to three components of the numerical demonstration: (i) pseudocount magnitude, (ii) effective read coverage, and (iii) the magnitude of the modeled wild-type-Peloria methylation contrast.

The purpose of this analysis was not to establish invariance of the information-theoretic quantities to biologically meaningful changes in methylation probability. Such invariance would be neither expected nor desirable, because entropy and divergence are specifically intended to respond to changes in the underlying distributions. Rather, the analysis was designed to distinguish *numerical sensitivity* of the absolute bit values from *structural robustness* of the principal interpretation of the demonstration, particularly the relative contribution of CG versus non-CG methylation.

For a cytosine i, with methylated-read count m_i and total read coverage n_i, the pseudocount-corrected methylation probability was calculated as

$$p_i(\alpha) = \frac{m_i + \alpha}{n_i + 2\alpha}.$$

For wild-type and peloric states, the corrected probabilities are denoted p_i and q_i, respectively. Site-specific Shannon entropy was calculated as

$$H(x) = -x\log_2 x - (1 - x)\log_2(1 - x),$$

and the total entropy of each state was obtained by summing across the 120 cytosines:

$$H_{\mathrm{WT}} = \sum_{i=1}^{120} H\,(p_i), \qquad H_{\mathrm{Pel}} = \sum_{i=1}^{120} H\,(q_i).$$

Directional Bernoulli KL divergence from Peloria to wild type was calculated as

$$D_{\mathrm{KL}}(q_i \| p_i) = q_i \log_2\left(\frac{q_i}{p_i}\right) + (1 - q_i)\log_2\left(\frac{1 - q_i}{1 - p_i}\right),$$

with regional divergence

$$D_{\mathrm{KL}}(\mathrm{Pel}\|\mathrm{WT}) = \sum_{i=1}^{120} D_{\mathrm{KL}}\,(q_i\|p_i).$$

For JSD, the midpoint probability was

$$r_i = \frac{p_i + q_i}{2},$$

and the site-specific bounded divergence was

$$\mathrm{JSD}_i = \frac{1}{2} D_{\mathrm{KL}}(p_i\|r_i) + \frac{1}{2} D_{\mathrm{KL}}(q_i\|r_i),$$

with

$$\mathrm{JSD}_{\mathrm{total}} = \sum_{i=1}^{120} \mathrm{JSD}_i.$$

All logarithms were calculated in base 2, and all information quantities are therefore expressed in bits.

S2.2. Sensitivity to pseudocount magnitude

To determine the influence of probability regularization, the original cytosine-level read counts were retained while the pseudocount was varied across

$$\alpha \in \{0.10,\ 0.25,\ 0.50,\ 1.00,\ 2.00\}.$$

The reference analysis corresponds to $\alpha = 0.50$. For each value of α, corrected wild-type and peloric methylation probabilities were recalculated at every cytosine, followed by recalculation of total entropy, directional KL divergence, summed site-wise Jensen–Shannon divergence, and the fraction of regional divergence attributable to non-CG (CHG + CHH) cytosines.

Supplementary Table S2A. Sensitivity to pseudocount magnitude.

Pseudocount α	H_{WT} (bits)	H_{Pel} (bits)	$D_{KL}(Pel\|WT)$ (bits)	JSD (bits)	Non-CG KL (%)	Non-CG JSD (%)
0.10	36.05	100.63	297.48	44.32	96.68	95.10
0.25	37.54	100.90	279.65	43.53	96.52	95.07
0.50	39.85	101.32	260.30	42.31	96.36	95.04
1.00	44.05	102.12	233.25	40.09	96.13	94.99
2.00	51.23	103.55	196.95	36.30	95.83	94.91

The absolute values showed different degrees of sensitivity to regularization. Peloric entropy changed only modestly over the tested range, whereas wild-type entropy increased as larger pseudocounts moved low methylation probabilities away from zero. Directional KL divergence was the most sensitive quantity, decreasing from 297.48 bits at $\alpha = 0.10$ to 196.95 bits at $\alpha = 2.00$. This behavior is expected because KL divergence responds strongly to probabilities near the boundaries of the Bernoulli interval. Summed site-wise Jensen–Shannon divergence showed a smaller change, from 44.32 to 36.30 bits.

Importantly, the context-level interpretation was highly stable. Across the entire pseudocount range, CHG and CHH cytosines together accounted for 95.83-96.68% of directional KL divergence and 94.91-95.10% of summed site-wise Jensen–Shannon divergence. Thus, regularization affected the absolute

numerical scale more strongly than the relative methylation-context architecture of the modeled epiallelic separation.

S2.3. Sensitivity to effective read coverage

To evaluate the effect of sequencing depth independently of the underlying site-specific methylation fractions, the original methylated-read fractions from the 50-read reference matrix were rescaled to effective coverages of

$$n \in \{10,\ 25,\ 50,\ 100,\ 200\}$$

reads per cytosine and per state. For each target coverage n, methylated-read counts were obtained by proportional rescaling of the reference counts and rounding to the nearest integer:

$$m_i^{(n)} = \mathrm{round}\left(n \frac{m_i^{(50)}}{50}\right).$$

The pseudocount was held fixed at $\alpha = 0.5$ for all coverage scenarios. This procedure preserves the site-specific methylation fractions as closely as permitted by integer read counts while changing the relative influence of the pseudocount. Small deviations at lower coverage therefore include the unavoidable discretization associated with integer read counts.

Supplementary Table S2B. Sensitivity to effective read coverage.

Coverage n (reads/site/state)	H_{WT} (bits)	H_{Pel} (bits)	$D_{KL}(Pel\|\|WT)$ (bits)	JSD (bits)	Non-CG KL (%)	Non-CG JSD (%)
10	48.94	104.27	207.57	36.69	96.11	95.06
25	40.95	101.93	257.32	41.45	96.39	95.05
50	39.85	101.32	260.30	42.31	96.36	95.04
100	37.54	100.90	279.65	43.53	96.52	95.07
200	36.30	100.68	293.72	44.18	96.64	95.09

Coverage affected the absolute values primarily through the relative weight of the fixed pseudocount. At low read depth, the pseudocount constitutes a larger fraction of the effective sample size and shifts site-specific probabilities toward 0.5; at higher depth, the observed methylated-read fraction exerts progressively greater influence. Consequently, directional KL divergence increased from 207.57 bits at 10 reads per site to 293.72 bits at 200 reads per site, whereas summed site-wise Jensen–Shannon divergence increased from 36.69 to 44.18 bits. Despite this numerical dependence, the context-specific interpretation was essentially unchanged. Non-CG cytosines accounted for 96.11-96.64% of total directional KL divergence and 95.04-95.09% of summed site-wise Jensen–Shannon divergence across the tested coverage range. Thus, the dominant role of CHG and CHH methylation in the synthetic demonstration did not depend materially on the assumed sequencing depth.

S2.4. Sensitivity to the modeled methylation contrast

The previous analyses alter numerical regularization or effective sampling depth while approximately preserving the underlying methylation architecture. We additionally tested whether the divergence measures respond in the expected direction when the magnitude of the modeled wild-type-Peloria contrast itself is changed.

Starting from the reference pseudocount-corrected probabilities ($\alpha = 0.5$), the peloric probability at each site was transformed according to

$$q_i^{(s)} = \mathrm{clip}[p_i + s(q_i - p_i),\ 0,\ 1],$$

where s is a global contrast-scaling parameter. Values of $s < 1$ reduce the separation between wild-type and peloric methylation probabilities, $s = 1$ reproduces the reference matrix, and $s > 1$ increases the separation. We evaluated

$$s \in \{0.80,\ 0.90,\ 1.00,\ 1.10\}.$$

For $s = 1.10$, two transformed peloric probabilities exceeded the Bernoulli upper bound and were therefore clipped to 1.0, as specified by the transformation above.

Supplementary Table S2C. Sensitivity to the magnitude of the modeled methylation contrast.

Contrast factor s	H_{WT} (bits)	H_{Pel} (bits)	$D_{KL}(Pel \| WT)$ (bits)	JSD (bits)
0.80	39.85	115.00	182.27	29.02
0.90	39.85	109.88	219.56	35.18
1.00	39.85	101.32	260.30	42.31
1.10	39.85	88.96	304.32	50.62

Both divergence measures responded monotonically to the imposed methylation contrast. Reducing the modeled separation to 80% of the reference contrast decreased directional KL divergence from 260.30 to 182.27 bits and JSD from 42.31 to 29.02 bits. Conversely, increasing the contrast by 10% increased the corresponding values to 304.32 and 50.62 bits. This behavior confirms that the divergence measures respond systematically to the magnitude of epiallelic separation rather than being tied to a single numerical realization of the synthetic matrix.

The entropy behavior illustrates a distinct property of the framework. Wild-type entropy remained fixed because p_i was unchanged, whereas peloric entropy decreased as the contrast was increased beyond the reference state. This is not paradoxical: Shannon entropy quantifies uncertainty, not methylation abundance. As probabilities approach either 0 or 1, methylation states become more predictable and entropy decreases. Thus, the contrast analysis also reinforces the conceptual distinction between methylation level, within-state entropy, and between-state divergence.

S2.5. Overall interpretation of the sensitivity analysis

Taken together, the sensitivity analyses show that the absolute bit values produced by the framework are not parameter-free. Directional KL divergence is particularly sensitive to pseudocount choice and effective read coverage when methylation probabilities approach 0 or 1, whereas summed site-wise Jensen–Shannon divergence is comparatively less affected. This dependence should be reported explicitly in empirical applications and argues against interpreting a single divergence value independently of sequencing depth and probability regularization.

At the same time, the principal structural conclusion of the synthetic demonstration was highly stable. Across all pseudocount values tested, non-CG methylation contributed approximately 95-97% of regional divergence, and essentially the same proportion was obtained across a 20-fold range of effective read coverage. The analysis therefore distinguishes sensitivity of the absolute numerical scale from robustness of the relative context-specific architecture. In addition, deliberate modification of the methylation contrast produced monotonic changes in KL divergence and JSD in the theoretically expected direction.

Accordingly, the sensitivity analysis supports use of the proposed framework as a comparative information-theoretic description of methylation-state differences, while emphasizing that empirical applications should report the underlying read depth, pseudocount or probability-estimation procedure, and relevant sensitivity analyses alongside absolute bit values.

Supplementary Section S3. Empirical application of the information-theoretic framework to publicly available cytosine-level bisulfite-PCR data from the *Arabidopsis thaliana FWA* locus

To complement the synthetic, literature-constrained Lcyc demonstration, we applied the same information-theoretic framework to an independent, publicly available experimental plant DNA-methylation dataset. We selected Arabidopsis thaliana data from Ghoshal et al. (2021), deposited in NCBI GEO under accession GSE149840. This dataset was chosen because it provides experimentally measured cytosine-level methylated and unmethylated read counts and includes an experimentally induced epigenetic transition at the FWA locus.

In that study, CRISPR-based MQ1 methyltransferase constructs were targeted to the FWA promoter to establish de novo DNA methylation, with corresponding wild-type, fwa, and catalytically inactive controls. Processed GEO files report, for each cytosine, genomic position, unmethylated-read count, methylated-read count, and percentage methylation, thereby providing exactly the count-level input required by the present framework.

S3.1. Rationale and experimental dataset

To complement the synthetic, literature-constrained *Lcyc* demonstration, we applied the same information-theoretic framework to an independent experimental plant DNA-methylation dataset for which cytosine-level methylated and unmethylated read counts are publicly available. We selected the *Arabidopsis thaliana FWA* targeted methylation dataset generated by Ghoshal et al. (2021) and deposited in NCBI Gene Expression Omnibus (GEO) under accession GSE149840. The study used CRISPR-based recruitment of the CG-specific bacterial DNA methyltransferase MQ1 to the *FWA* promoter in an *fwa* epimutant background. The experimental design included a catalytically inactive dMQ1 control and active MQ1v constructs, providing a biologically interpretable contrast between a control methylation state and experimentally induced DNA methylation at the same locus.

For this empirical demonstration, we analyzed the targeted bisulfite-PCR (BSPCR) samples GSM4514077 (dMQ1 T1-1; control) and GSM4514078 (MQ1v T1-7; active methyltransferase). Both samples were derived from rosette leaves in a Col-0, *fwa* genetic background and were sequenced after bisulfite treatment. GEO provides processed BED-like files separately for CG, CHG, and CHH contexts. Each record contains chromosome, cytosine genomic coordinate, coordinate + 1, the number of unmethylated reads, the number of methylated reads, and the percentage of methylated reads. Therefore, unlike the synthetic *Lcyc* illustration, this analysis uses directly observed experimental read counts and naturally heterogeneous site-specific coverage.

The purpose of this analysis is methodological: to demonstrate that the framework can be applied without modification to real cytosine-level bisulfite-sequencing counts. It is not intended to establish a general biological estimate for *FWA*, nor to substitute *FWA* for *Lcyc* as the motivating case study.

S3.2. Cytosine matching, coverage filtering, and probability estimation

Cytosines were matched between GSM4514077 and GSM4514078 by chromosome and genomic coordinate within each methylation context. Only cytosines observed in both samples and covered by at least five reads in each condition were retained. This conservative filter excluded sites at which a methylation fraction would be determined by only one or a few reads. After filtering, 79 shared cytosines were retained: 16 CG, 5 CHG, and 58 CHH sites.

For cytosine i and condition X, the total coverage was defined as

$$n_i^{(X)} = m_i^{(X)} + u_i^{(X)},$$

where $m_i^{(X)}$ and $u_i^{(X)}$ denote the experimentally observed methylated and unmethylated read counts, respectively. To retain the same regularization convention used in the *Lcyc* demonstration and to avoid exact probabilities of 0 or 1, the methylation probability was estimated with a Jeffreys-type pseudocount $\alpha = 0.5$ as

$$p_i^{(X)} = \frac{m_i^{(X)} + \alpha}{n_i^{(X)} + 2\alpha}, \qquad \alpha = 0.5.$$

We denote the corrected dMQ1 control probability by p_i and the corrected MQ1v probability by q_i.

S3.3. Information-theoretic quantities

S3.3.1. Maximum binary capacity

Under the binary-state representation used throughout the framework, each retained cytosine contributes at most one bit of formal binary capacity. For N shared cytosines,

$$C_{\max} = N \text{ bits}.$$

Thus, for the 79-site empirical matrix used here, $C_{\max} = 79$ bits. This is a formal upper bound and should not be interpreted as observed entropy.

S3.3.2. Shannon entropy

For a Bernoulli methylation probability r, site-specific Shannon entropy was calculated as

$$H(r) = -r\log_2 r - (1-r)\log_2(1-r).$$

Regional entropy for each experimental condition was obtained by summing the site-specific values over the shared cytosines:

$$H_{\text{dMQ1}} = \sum_{i=1}^{N} H\,(p_i), \qquad H_{\text{MQ1v}} = \sum_{i=1}^{N} H\,(q_i).$$

As in the principal framework, this additive regional entropy is a first-order measure that assumes site-wise independence.

S3.3.3. Directional Kullback-Leibler divergence

The directional divergence of the active MQ1v methylation state relative to the dMQ1 control was calculated as

$$D_{\text{KL}}(q_i \| p_i) = q_i \log_2\left(\frac{q_i}{p_i}\right) + (1-q_i)\log_2\left(\frac{1-q_i}{1-p_i}\right),$$

with the regional total

$$D_{\text{KL}}(\text{MQ1v}\|\text{dMQ1}) = \sum_{i=1}^{N} D_{\text{KL}}\,(q_i \| p_i).$$

S3.3.4. Symmetric divergence and summed site-wise Jensen–Shannon divergence

For site ranking, symmetric KL divergence was defined as

$$D_{i,\text{sym}} = \frac{1}{2}[D_{\text{KL}}(q_i \| p_i) + D_{\text{KL}}(p_i \| q_i)].$$

For the summed site-wise Jensen–Shannon divergence, the midpoint probability was

$$r_i = \frac{p_i + q_i}{2},$$

and the site-specific Jensen-Shannon divergence was

$$JSD_i = \frac{1}{2}D_{\mathrm{KL}}(p_i \| r_i) + \frac{1}{2}D_{\mathrm{KL}}(q_i \| r_i).$$

The regional bounded divergence was

$$JSD_{\mathrm{total}} = \sum_{i=1}^{N} JSD_i.$$

Because each site is Bernoulli, $0 \leq JSD_i \leq 1$ bit, and therefore $JSD_{\mathrm{total}} \leq N$ bits.

S3.3.5. Empirical dimensionality

To quantify how distributed the experimental transition was across cytosines, sites were ranked by $D_{i,\mathrm{sym}}$. The fractional contribution of site i was

$$\rho_i = \frac{D_{i,\mathrm{sym}}}{\sum_{j=1}^{N} D_{j,\mathrm{sym}}},$$

and the cumulative fraction after the first k ranked sites was

$$R(k) = \sum_{j=1}^{k} \rho_{(j)},$$

where $\rho_{(j)}$ denotes the jth largest contribution. The numbers of sites required to reach 50%, 80%, and 90% of total symmetric divergence were recorded as operational dimensionality descriptors.

S3.4. Empirical results

The experimental matrix contained 79 cytosines shared between the two BSPCR samples after the coverage filter. Across all retained sites, the mean corrected methylation probability increased from 0.00137 in the dMQ1 control to 0.04063 in MQ1v. This overall mean is strongly influenced by the predominance of CHH sites in the retained matrix; context-specific analysis is therefore more informative.

S3.4.1. Context-specific methylation and entropy

The largest experimental change occurred in the CG context, consistent with the CG specificity of MQ1. Mean corrected CG methylation increased from 0.00095 in the dMQ1 control to 0.15945 in MQ1v. In contrast, CHG and CHH methylation remained much lower, with mean corrected MQ1v probabilities of 0.01294 and 0.01023, respectively.

The total Shannon entropy increased from 1.10 bits in the dMQ1 control to 13.03 bits in MQ1v across all 79 cytosines. Most of this increase arose from the 16 shared CG sites, for which entropy increased from 0.17 to 8.50 bits. This empirical result illustrates the distinction between methylation abundance and entropy: the increased entropy reflects movement of several CG probabilities away from near-zero methylation toward intermediate methylation fractions, rather than an intrinsic informational property of methylation itself.

S3.4.2. Experimental divergence

The total directional divergence $D_{\mathrm{KL}}(\mathrm{MQ1v}\|\mathrm{dMQ1})$ was 18.94 bits, and the summed site-wise Jensen–Shannon divergence was 1.58 bits. CG cytosines contributed 17.34 bits (91.6%) of directional KL divergence and 1.37 bits (86.9%) of total JSD. CHG contributed only 0.03 bits of KL and 0.006 bits of JSD, while CHH contributed 1.57 bits of KL and 0.20 bits of JSD. Thus, the information-theoretic decomposition correctly identifies the experimentally targeted CG context as the dominant contributor to separation between the active-MQ1v and inactive-dMQ1 states.

S3.4.3. Dimensionality of the experimental transition

Ranking the 79 cytosines by symmetric KL contribution showed that 4 sites accounted for 50% of total symmetric divergence, 8 sites accounted for 80%, and 12 sites accounted for 90%. Nine of the top 10 ranked cytosines were CG sites. These results indicate that, in this targeted experimental comparison, most of the information-theoretic separation is concentrated in a relatively small subset of cytosines, in marked contrast to the deliberately distributed synthetic architecture used for the *Lcyc* methodological demonstration. This contrast illustrates that the dimensionality metric can distinguish concentrated from broadly distributed methylation changes rather than merely returning a fixed property of the computational procedure.

Supplementary Table S3. Summary of the empirical BSPCR information-theoretic analysis

Context	Shared sites (N)	Mean p dMQ1	Mean q MQ1v	H dMQ1 (bits)	H MQ1v (bits)	KL(MQ1v ‖ dMQ1) (bits)	Summed site-wise JSD (bits)	Symmetric KL, D_sym (bits)
CG	16	0.00095	0.15945	0.17	8.50	17.34	1.373	10.77
CHG	5	0.00600	0.01294	0.23	0.45	0.03	0.006	0.02
CHH	58	0.00108	0.01023	0.70	4.09	1.57	0.202	1.09
Total	79	0.00137	0.04063	1.10	13.03	18.94	1.581	11.89

Note: Probabilities were estimated from experimentally observed methylated and unmethylated read counts using $\alpha = 0.5$. Only cytosines shared by both samples and covered by at least five reads in each sample were retained. The "Total" mean probability is an unweighted mean across retained cytosines, whereas entropy and divergence quantities are sums of site-specific values.

Supplementary Table S3A. Cytosine-level results for the experimentally targeted CG context

TAIR10 position (0-based)	dMQ1 coverage	dMQ1 methylated reads	MQ1v coverage	MQ1v methylated reads	p dMQ1	q MQ1v	KL(MQ1v ‖ dMQ1) (bits)	Site-wise JSD (bits)
13038249	58481	66	24919	9917	0.00114	0.39797	2.9236	0.2298
13038268	58480	53	24922	8671	0.00091	0.34793	2.5807	0.1963
13038271	58478	54	24921	9819	0.00093	0.39401	3.0001	0.2278
13038356	55634	76	25826	6516	0.00138	0.25231	1.5851	0.1337
13038360	55638	47	25812	5667	0.00085	0.21956	1.4798	0.1159
13038363	55627	29	25817	6933	0.00053	0.26855	2.0833	0.1468
13038384	55640	75	25820	2550	0.00136	0.09878	0.4775	0.0467
13038467	83029	74	42260	1796	0.00090	0.04251	0.1778	0.0189
13038475	83049	94	42265	839	0.00114	0.01986	0.0552	0.0074
13038567	76036	104	43398	2707	0.00137	0.06239	0.2581	0.0278
13038574	76051	62	43418	7420	0.00082	0.17090	1.0928	0.0878
13038581	76051	47	43423	6343	0.00062	0.14608	0.9558	0.0746
13038595	76050	54	43424	2315	0.00072	0.05332	0.2577	0.0248
13038605	76035	25	43410	2738	0.00034	0.06308	0.3890	0.0309
13038677	92195	88	51926	550	0.00096	0.01060	0.0229	0.0034
13038685	92132	114	51890	175	0.00124	0.00338	0.0018	0.0004

S3.5. Interpretation and limitations

This independent empirical example addresses a key methodological question raised by the synthetic *Lcyc* illustration: whether the proposed calculations can be applied to real methylation data with

nonuniform sequencing depth and experimentally observed count distributions. The answer is affirmative. No modification of the framework was required: observed methylated and unmethylated counts were converted to corrected Bernoulli probabilities and then to entropy, directional divergence, site-wise symmetric divergence, context decomposition, and dimensionality measures using the same equations as in the principal analysis.

The empirical example also provides a useful positive control for biological interpretability. Because MQ1 is CG-specific, the active MQ1v condition is expected to produce its principal methylation effect in the CG context. The information-theoretic decomposition recovers exactly this structure: CG sites account for more than 90% of directional KL divergence and nearly 87% of JSD in the filtered experimental matrix. Thus, the framework does not depend on the particular synthetic assumptions used for *Lcyc* and can identify the experimentally targeted methylation context from real cytosine-level data.

Several limitations should nevertheless be emphasized. First, the comparison uses one dMQ1 BSPCR sample and one MQ1v BSPCR sample and is therefore a proof-of-application rather than an inferential population-level analysis. Second, BSPCR coverage is extremely high at many amplified positions and heterogeneous across sites; the pseudocount consequently has negligible influence at highly covered cytosines but a larger effect near the coverage threshold. Third, the additive entropy and divergence totals retain the site-independence assumption of the main framework. Finally, the *FWA* experiment is biologically distinct from the naturally occurring peloric *Lcyc* epiallele. It is included solely to demonstrate empirical applicability of the general method, while *Lcyc* remains the motivating case study for the conceptual framework.

S3.6. Reproducibility statement
The analysis used the publicly deposited processed files:
GSM4514077_P1_T1_1_CpG.bed.gz,
GSM4514077_P1_T1_1_CHG.bed.gz,
GSM4514077_P1_T1_1_CHH.bed.gz,
GSM4514078_P1_T1_7_CpG.bed.gz,
GSM4514078_P1_T1_7_CHG.bed.gz,
and GSM4514078_P1_T1_7_CHH.bed.gz, from GEO series GSE149840. Coordinates correspond to TAIR10. Cytosines were paired by chromosome and 0-based cytosine coordinate, filtered at a minimum coverage of five reads in both samples, and analyzed using base-2 logarithms and pseudocount $\alpha = 0.5$. The resulting 79-site matrix consisted of 16 CG, 5 CHG, and 58 CHH cytosines.

Supplementary Note S4. Effect of inter-cytosine dependence on the site-wise information approximation

S4.1. Rationale
The first-order framework developed in the main manuscript represents each assayed cytosine as a Bernoulli random variable and summarizes regional methylation information by summing site-specific quantities. This construction is intentionally transparent and is compatible with standard cytosine-level bisulfite-sequencing outputs, which generally provide methylated and unmethylated read counts at individual genomic positions. However, the additive formulation is equivalent to a factorized approximation in which methylation states at different cytosines are treated as statistically independent.

Real DNA-methylation patterns need not satisfy this assumption. Neighboring cytosines can show coordinated methylation because of local chromatin state, maintenance methyltransferase activity,

cell-type composition, allelic state, clonal structure, or epihaplotype organization. Consequently, the site-wise framework should be interpreted as a first-order description of *marginal* cytosine methylation probabilities rather than as a complete description of the joint molecular methylation state.

This Supplementary Note formalizes the consequences of that approximation for (i) Shannon entropy, (ii) Kullback-Leibler and Jensen-Shannon divergence, and (iii) the operational dimensionality measure used in the manuscript. It also illustrates, with simple two-cytosine examples, the distinction between information contained in marginal methylation frequencies and information contained in dependencies among cytosines.

S4.2. Site-wise entropy and joint methylation entropy

Let $X_i \in \{0,1\}$ denote the methylation state of cytosine i, where $X_i = 1$ represents methylation and $X_i = 0$ represents the unmethylated state. If the marginal methylation probability at cytosine i is $p_i = P(X_i = 1)$, the Bernoulli entropy used in the main framework is

$$H(X_i) = -p_i \log_2 p_i - (1 - p_i) \log_2 (1 - p_i).$$

The current regional entropy is calculated as the sum of the marginal entropies:

$$H_{\text{sitewise}} = \sum_{i=1}^{N} H\,(X_i).$$

The complete information-theoretic description of the regional methylation pattern would instead require the joint distribution of all cytosine states,

$$P(X_1, X_2, \dots, X_N),$$

and its corresponding joint entropy,

$$H_{\text{joint}} = H(X_1, X_2, \dots, X_N) = -\sum_{\mathbf{x}} P\,(\mathbf{x}) \log_2 P(\mathbf{x}),$$

where the sum extends over all 2^N possible binary methylation patterns $\mathbf{x} = (x_1, \dots, x_N)$.

A fundamental entropy inequality gives

$$H(X_1, X_2, \dots, X_N) \leq \sum_{i=1}^{N} H\,(X_i),$$

with equality if and only if the cytosine states are statistically independent. Therefore,

$$H_{\text{joint}} \leq H_{\text{sitewise}}.$$

The summed site-wise entropy should consequently be interpreted as an independence-based upper estimate of the true joint methylation entropy. When neighboring cytosines are correlated, part of the uncertainty counted separately at each site is redundant at the regional level.

The magnitude of this dependence can be quantified by the *total correlation* (also termed multi-information):

$$TC(X_1, \dots, X_N) = \sum_{i=1}^{N} H\,(X_i) - H(X_1, \dots, X_N).$$

Throughout this study, information is used in the Shannon-theoretic statistical sense. Entropy and divergence quantify uncertainty and statistical distinguishability of methylation-state distributions; they do not measure functional biological information or causal contribution to floral morphology. Functional interpretation requires independent experimental evidence linking methylation configurations to gene regulation, chromatin state, developmental context, and phenotype. Consider two cytosines, X_1 and X_2, each with marginal methylation probability $P(X_i = 1) = 0.5$. Each site therefore has maximal Bernoulli entropy:

$$H(X_1) = H(X_2) = 1 \text{ bit}.$$

The site-wise entropy is

$$H_{\text{sitewise}} = H(X_1) + H(X_2) = 2 \text{ bits}.$$

Now suppose that the two cytosines are perfectly correlated and occur only in the patterns 00 and 11, each with probability 0.5:

Molecular methylation pattern	Probability
00	0.5
11	0.5
01	0
10	0

The joint entropy is then

$$H(X_1, X_2) = 1 \text{ bit},$$

and therefore

$$TC = 2 - 1 = 1 \text{ bit}.$$

Although two cytosines are observed, they contain only one independent binary degree of uncertainty because the state of one cytosine completely predicts the state of the other. This example shows why summing marginal entropies can overestimate the effective number of independent methylation-state degrees of freedom in strongly correlated regions.

S4.4. Divergence under the factorized approximation

The same conceptual distinction applies to epiallelic divergence. Let P denote the joint methylation distribution in one state (for example, Peloria) and Q the joint distribution in the comparison state (for example, wild type). The complete joint Kullback-Leibler divergence would be

$$D_{KL}(P \| Q) = \sum_{\mathbf{x}} P\,(\mathbf{x}) \log_2 \frac{P(\mathbf{x})}{Q(\mathbf{x})}.$$

In the current framework, only the marginal Bernoulli distributions P_i and Q_i are used. The regional KL divergence is calculated as

$$D_{KL}^{\text{sitewise}}(P\|Q) = \sum_{i=1}^{N} D_{KL}\,(P_i\|Q_i).$$

This expression is exactly the KL divergence between the corresponding product-distribution approximations,

$$P_{\text{ind}}(\mathbf{x}) = \prod_{i=1}^{N} P_i\,(x_i), \qquad Q_{\text{ind}}(\mathbf{x}) = \prod_{i=1}^{N} Q_i\,(x_i),$$

because

$$D_{KL}(\prod_i P_i \parallel \prod_i Q_i) = \sum_i D_{KL}\,(P_i\|Q_i).$$

Therefore, the summed site-specific KL divergence should be interpreted as the divergence between *factorized, site-independent Bernoulli approximations* of the two methylation profiles. It measures differences in marginal methylation probabilities and does not, by itself, capture differences that reside exclusively in inter-site dependence structure.

An analogous interpretation applies to the site-wise Jensen-Shannon divergence. The quantity used in the manuscript,

$$JSD_{\text{sitewise}} = \sum_{i=1}^{N} JSD(P_i, Q_i),$$

provides a bounded, symmetric summary of marginal site-level separation. It should not be equated with the Jensen-Shannon divergence between complete multivariate epihaplotype distributions unless the relevant factorization assumptions hold.

Unlike the entropy case, the summed marginal KL or Jensen-Shannon divergence is not generally described as a universal upper or lower bound on the corresponding divergence between arbitrary biological joint distributions. Its most rigorous interpretation is therefore as a divergence computed within the factorized approximation.

S4.5. Two-cytosine example: identical marginals but different epihaplotype structure

Marginal methylation frequencies can fail to detect differences that are encoded entirely in cytosine-cytosine dependencies. Consider two epigenetic states with the following molecular patterns:

State A

Pattern	Probability
00	0.5
11	0.5
01	0
10	0

State B

Pattern	Probability
01	0.5
10	0.5

Pattern	Probability
00	0
11	0

For both states,

$$P(X_1 = 1) = P(X_2 = 1) = 0.5.$$

Thus, every individual cytosine has the same marginal methylation probability in State A and State B. A purely site-wise comparison therefore gives

$$D_{KL}^{\text{sitewise}} = 0, \qquad JSD_{\text{sitewise}} = 0.$$

Nevertheless, the molecular methylation patterns are entirely different: State A contains concordant epihaplotypes (00 and 11), whereas State B contains discordant epihaplotypes (01 and 10). A joint-state analysis would detect this distinction immediately.

This example illustrates an important interpretive boundary of the current framework: it quantifies information contained in *marginal cytosine methylation probabilities*, whereas information encoded solely in the *relationships among cytosines* requires a joint or epihaplotype-level analysis.

S4.6. Biological sources of inter-cytosine dependence

Several biological and experimental mechanisms can generate dependence among methylation states. These include coordinated activity of maintenance and de novo methylation pathways, spreading or domain-level chromatin regulation, local sequence context, allele-specific methylation, clonal or lineage structure, cell-type composition, and molecule-specific epihaplotype organization. Consequently, an intermediate methylation probability at a single cytosine can arise from biologically distinct underlying structures.

For example, an observed marginal probability of $p = 0.5$ may reflect: (i) stochastic methylation of individual molecules, (ii) a mixture of fully methylated and fully unmethylated cells, (iii) allele-specific methylation, or (iv) a mixture of cell types with different regional methylation states. These scenarios may have similar site-wise entropy but very different joint methylation architectures.

Accordingly, site-specific entropy should be interpreted as uncertainty in the methylation state of a randomly sampled observation at that cytosine. It does not, on its own, distinguish whether the uncertainty arises within molecules, between cells, between alleles, or between biological subpopulations.

S4.7. Consequences for the dimensionality analysis

The dimensionality analysis in the main manuscript ranks cytosines according to their site-specific contribution to total symmetric divergence and determines the number of sites required to explain a specified cumulative fraction of the summed divergence. Let d_i denote the selected site-specific symmetric divergence and define

$$\rho_i = \frac{d_i}{\sum_{j=1}^{N} d_j}.$$

After ranking sites in decreasing order of ρ_i, the cumulative contribution of the first k sites is

$$R(k) = \sum_{i=1}^{k} \rho_{(i)}.$$

The threshold-based dimensionality statistic is

$$N_\tau = \min\{k: R(k) \geq \tau\},$$

for thresholds such as $\tau = 0.5$, 0.8, or 0.9.

Under the independence-based framework, N_τ should be interpreted as a measure of *site-wise divergence dimensionality*. It quantifies how many cytosine positions are required to account for a chosen fraction of the summed marginal divergence. It is not necessarily equal to the number of statistically independent epigenetic degrees of freedom.

For example, if 20 neighboring cytosines belong to a tightly coordinated methylation block, the site-wise analysis may identify all 20 as important contributors even though the underlying regulatory change may be governed by substantially fewer effective molecular dimensions. Conversely, a joint-state analysis may reveal combinatorial differences that are invisible in marginal site rankings.

For this reason, the terms low-, intermediate-, and high-dimensional used in the present framework are operational descriptors of the distribution of *site-wise* divergence and should not be interpreted as definitive estimates of latent biological dimensionality.

S4.8. Extension to dependency-aware information measures

The present formulation was designed as a first-order framework because cytosine-level count data are widely available and permit transparent, reproducible estimation of marginal methylation probabilities. When single-molecule or sufficiently resolved epihaplotype data are available, the framework can be extended to dependency-aware measures.
Potential extensions include:

1. Joint entropy, $H(X_1, \ldots, X_N)$, to quantify the uncertainty of complete regional methylation patterns.
2. Pairwise mutual information,

$$I(X_i; X_j) = H(X_i) + H(X_j) - H(X_i, X_j),$$

to quantify statistical dependence between pairs of cytosines. 3. **Total correlation**,

$$TC = \sum_i H(X_i) - H(X_1, \ldots, X_N),$$

to quantify the aggregate dependency or redundancy across a region. 4. **Conditional entropy**, such as $H(X_i \mid X_j)$, to determine how uncertainty at one site changes after observing another. 5. **Joint-state KL or Jensen-Shannon divergence**, calculated directly from empirical epihaplotype distributions, to compare entire methylation-pattern architectures rather than only marginal site probabilities. 6. **Block- or domain-level dimensionality**, in which correlated cytosines are grouped into effective units before calculating cumulative divergence.

Such extensions require substantially richer data than conventional site-wise methylation summaries. In particular, reliable estimation of a joint distribution over many binary sites is statistically demanding because the number of possible epihaplotypes grows as 2^N. Long-read bisulfite sequencing, nanopore-based methylation profiling, or other single-molecule approaches can reduce this limitation by directly preserving co-methylation information across multiple sites on the same molecule.

S4.9. Interpretation within the present study

The numerical quantities reported for the synthetic *Lcyc* demonstration and the empirical *FWA* example should therefore be interpreted at the level at which they are calculated. The reported entropies are summed marginal Bernoulli entropies; the reported KL and Jensen-Shannon values quantify separation between site-wise methylation-probability profiles; and the dimensionality statistics describe the concentration or dispersion of those site-wise divergence contributions.

These measures remain useful because they provide a reproducible first-order language for comparing cytosine-level methylation profiles using data formats that are routinely generated by bisulfite-sequencing analyses. Their principal limitation is not that the calculations are mathematically ill-defined, but that they intentionally omit dependency information that may be biologically meaningful. Accordingly, the current and future versions of the framework can be conceptually separated as

current framework: marginal methylation information

and

future joint-state extension: marginal information + dependency information.

This distinction defines the scope of the present measurements and provides a natural path for extending the framework when molecule-resolved methylation data become available.

S4.10. Summary statement

In summary, summing site-specific information quantities constitutes a transparent first-order approximation in which cytosines are represented as independent Bernoulli variables. For entropy, this produces an upper estimate of joint methylation entropy, with the discrepancy determined by inter-site dependency or total correlation. For KL and Jensen-Shannon divergence, the summed site-specific values quantify separation between factorized marginal approximations and may miss differences encoded exclusively in co-methylation or epihaplotype structure. The current dimensionality analysis should likewise be interpreted as site-wise rather than as a direct estimate of independent molecular degrees of freedom. These limitations define the intended scope of the present framework and motivate future extensions based on joint entropy, mutual information, total correlation, and joint-state divergence.

Supplementary Note S5. Precise definition and interpretation of the summed site-wise Jensen-Shannon divergence

The following supplementary notes complement Supplementary Section S2 (sensitivity analysis), Supplementary Section S3 (empirical FWA demonstration), and Supplementary Note S4 (inter-cytosine dependence).

S5.1. Site-specific Bernoulli distributions

For each cytosine i, methylation in the wild-type and peloric states is represented by Bernoulli distributions with corrected methylation probabilities p_i and q_i, respectively:

$$P_i = \text{Bernoulli}(p_i), \quad Q_i = \text{Bernoulli}(q_i).$$

Using equal weights for the two states, the midpoint Bernoulli distribution is defined by the probability

$$m_i = (p_i + q_i)/2, \quad M_i = \text{Bernoulli}(m_i).$$

S5.2. Site-specific Jensen-Shannon divergence

The Jensen-Shannon divergence at cytosine i is defined as

$$JSD_i(P_i,Q_i) = \frac{1}{2} D\_KL(P_i \parallel M_i) + \frac{1}{2} D\_KL(Q_i \parallel M_i),$$

where all logarithms are base 2. For a Bernoulli variable, this can be written explicitly as

$$D\_KL(P_i \parallel M_i) = p_i \log_2(p_i/m_i) + (1-p_i) \log_2[(1-p_i)/(1-m_i)],$$

$$D\_KL(Q_i \parallel M_i) = q_i \log_2(q_i/m_i) + (1-q_i) \log_2[(1-q_i)/(1-m_i)].$$

Because JSD_i is the conventional equal-weight Jensen-Shannon divergence between two Bernoulli distributions, each site-specific contribution satisfies

$$0 \leq JSD_i \leq 1 \text{ bit}.$$

S5.3. Regional additive statistic

The quantity reported for the complete assayed region is not a single conventional Jensen-Shannon divergence between two full regional joint distributions. It is the additive sum of the site-specific Bernoulli divergences:

$$JSD\_\Sigma = \Sigma_{i=1}^{N} JSD_i.$$

Therefore, for N cytosines,

$$0 \leq JSD\_\Sigma \leq N \text{ bits}.$$

For the 120-cytosine synthetic Lcyc demonstration, the formal upper bound is therefore 120 bits. The value 42.31 bits reported for the reference demonstration should be interpreted as a summed site-wise Jensen-Shannon divergence, not as a conventional regional JSD bounded by 1 bit.

S5.4. Relation to product distributions and joint methylation structure

This distinction is important because Jensen-Shannon divergence is not additive over independent components in the same way as Kullback-Leibler divergence. Even if regional states were represented by product Bernoulli distributions, JSD_Σ should not in general be identified with the single Jensen-Shannon divergence between those product distributions. Instead, JSD_Σ is an intentionally additive marginal summary that assigns an interpretable bounded contribution to each assayed cytosine.

Consequently, JSD_Σ measures how strongly the two states differ when examined cytosine by cytosine. It does not capture separation that is encoded exclusively in correlations or epihaplotype structure. The consequences of such dependencies are treated formally in Supplementary Note S4.

S5.5. Terminological recommendation

To avoid ambiguity, the revised manuscript should use the expression “summed site-wise Jensen-Shannon divergence (JSD_Σ)” at first mention and “site-wise JSD” thereafter. Statements that JSD_Σ is “bounded” should specify that the 1-bit bound applies to each Bernoulli site, whereas the additive regional statistic is bounded by N bits.

Supplementary Note S6. Statistical information versus functional biological information

S6.1. Meaning of information in the present framework

The term information is used throughout the framework in the Shannon-theoretic statistical sense. It refers to uncertainty, predictability, and distinguishability of probability distributions. The quantities

defined here do not, by themselves, measure biological function, regulatory meaning, adaptive value, semantic content, or causal contribution to phenotype.
At a single cytosine, Shannon entropy

$$H(p_i) = -p_i \log_2 p_i - (1-p_i) \log_2(1-p_i)$$

quantifies uncertainty in methylation state. A site with p_i close to 0 or 1 has low entropy because its state is predictable, whereas a site with p_i close to 0.5 has high entropy because methylated and unmethylated observations are similarly probable. Neither situation is intrinsically more biologically important.

S6.2. Statistical distinguishability is not causal effect

Likewise, KL divergence and JSD_Σ quantify statistical separation between methylation-state descriptions. A large contribution from a cytosine means that its methylation probability differs strongly between the compared states under the chosen model. It does not imply that the cytosine is necessary or sufficient for the phenotype, nor that the corresponding number of bits represents the information required to generate the phenotype.
Functional interpretation requires independent biological evidence, including effects on transcription, chromatin organization, developmental timing, tissue specificity, allele-specific regulation, or phenotype. Thus, the framework should be viewed as quantifying the statistical architecture of an epigenetic difference, while causal or functional claims must be established by experimental biology.

S6.3. Why the distinction matters for Lcyc

The peloric Lcyc system provides a biologically compelling case because methylation of the locus is associated with transcriptional silencing and altered floral symmetry. However, the numerical bit values obtained from the synthetic, literature-constrained demonstration characterize the constructed methylation distributions. They are not measurements of "functional information" contained in the flower, nor should the peloric phenotype be described as being caused by a specific number of bits.
A more precise interpretation is that the framework quantifies: (i) the formal binary capacity of the assayed cytosines; (ii) uncertainty within each modeled methylation state; (iii) marginal statistical distinguishability between states; (iv) context-specific contributions; and (v) how broadly site-wise divergence is distributed across the locus.

S6.4. Relationship to the empirical FWA demonstration

The empirical FWA analysis reinforces the same distinction. The information-theoretic quantities summarize experimentally observed differences in cytosine-level methylation counts. The biological interpretation of those changes derives from the experimental context of FWA regulation, not from entropy or divergence values alone. The statistical framework and the functional biology are therefore complementary rather than interchangeable levels of description.

Supplementary Note S7. Robustness of site-wise dimensionality to operational definitions

S7.1. Operational definition used in the framework

The dimensionality analysis asks whether cumulative site-wise divergence is concentrated in a small number of cytosines or broadly distributed across the assayed region. Let $d_i \geq 0$ denote the chosen site-specific divergence contribution and define the normalized contribution weight

$$\rho_i = d_i / \Sigma_j d_j, \quad \text{with} \quad \Sigma_i \rho_i = 1.$$

After ranking the weights in descending order, $\rho_{(1)} \geq \rho_{(2)} \geq \ldots \geq \rho_{(N)}$, the cumulative contribution of the top k sites is

$$R(k) = \Sigma_{i=1}^{k} \rho_{(i)}.$$

For a chosen threshold τ, the corresponding site count is

$$N_\tau = \min\{k : R(k) \geq \tau\}.$$

These quantities define a site-wise divergence dimensionality. They should not be interpreted as the number of statistically independent epigenetic degrees of freedom; correlated sites can represent fewer effective molecular dimensions, as discussed in Supplementary Note S4.

S7.2. Sensitivity to cumulative threshold and site-ranking metric

To assess whether the dimensionality conclusion depends strongly on a particular operational choice, the 120-site synthetic matrix was recalculated using two site-ranking measures: symmetric KL divergence and site-wise JSD. Multiple cumulative thresholds were examined rather than relying on a single 80% or 90% cutoff.

Supplementary Table S7A. Number of ranked cytosines required to reach each cumulative divergence threshold under symmetric-KL and site-wise-JSD ranking.

Cumulative threshold	Symmetric-KL ranking	Site-wise-JSD ranking
50%	38	40
70%	59	61
80%	72	74
90%	88	90
95%	97	99

The classification is therefore not tied to one threshold or one ranking measure. Under both definitions, dozens of sites are needed to explain half of the cumulative divergence, and approximately 72-90 sites are required to explain 80-90% of the divergence. The central conclusion - that the synthetic transition is broadly distributed rather than dominated by only a few cytosines - is preserved.

S7.3. Threshold-free effective numbers of contributing sites

As a complementary threshold-free description, effective numbers of contributing sites can be calculated from the normalized weights. The inverse-Simpson effective number is

$$N_{eff,2} = 1 / \Sigma_i \rho_i^2,$$

and the Shannon effective number is

$$N_{eff,1} = 2^{[-\Sigma_i \rho_i \log_2 \rho_i]}.$$

These quantities can be interpreted as the number of equally contributing sites that would produce the same concentration of weights. For the synthetic 120-site matrix, the recalculated values are:

Supplementary Table S7B. Threshold-free effective numbers of contributing sites.

Effective-site metric	Symmetric KL	Site-wise JSD
Inverse-Simpson effective number, $N_{eff,2}$	93.6	97.6

Effective-site metric	Symmetric KL	Site-wise JSD
Shannon effective number, N_eff,1	102.1	104.6

Both threshold-free measures indicate a high effective number of contributing sites. This is consistent with the cumulative-threshold analysis and supports an intermediate-to-high site-wise dimensionality under the assumptions of the demonstration matrix.

S7.4. Recalculation and reproducibility clarification

Recalculation of the dimensionality analysis directly from the cytosine-level values revealed a small inconsistency between the originally stated threshold counts and the values obtained using the ranking definition specified in Methods. Using symmetric KL contributions, the corrected counts are 38 sites for 50%, 72 sites for 80%, and 88 sites for 90%. Using site-wise JSD contributions, the corresponding values are 40, 74, and 90 sites. This numerical correction does not change the qualitative interpretation, but it should be propagated consistently to the Results, Figure 4, its legend, and the Supplementary Information.

S7.5. Recommended interpretation

The dimensionality labels low, intermediate, and high should be treated as operational descriptors rather than universal biological categories. Their interpretation depends on locus size, sequencing depth, site filtering, correlation structure, and the divergence measure used for ranking. Accordingly, empirical applications should preferably report the complete cumulative contribution curve together with one or more threshold-free effective-site measures, rather than relying on a single cutoff.
